# GraphBNC: Machine Learning‐Aided Prediction of Interactions Between Metal Nanoclusters and Blood Proteins

**DOI:** 10.1002/adma.202407046

**Published:** 2024-09-24

**Authors:** Antti Pihlajamäki, María Francisca Matus, Sami Malola, Hannu Häkkinen

**Affiliations:** ^1^ Department of Physics Nanoscience Center University of Jyväskylä Jyväskylä FI‐40014 Finland; ^2^ Department of Chemistry Nanoscience Center University of Jyväskylä Jyväskylä FI‐40014 Finland

**Keywords:** graphs, machine learning, metal nanoclusters, molecular dynamics, nano–bio interface

## Abstract

Hybrid nanostructures between biomolecules and inorganic nanomaterials constitute a largely unexplored field of research, with the potential for novel applications in bioimaging, biosensing, and nanomedicine. Developing such applications relies critically on understanding the dynamical properties of the nano–bio interface. This work introduces and validates a strategy to predict atom‐scale interactions between water‐soluble gold nanoclusters (AuNCs) and a set of blood proteins (albumin, apolipoprotein, immunoglobulin, and fibrinogen). Graph theory and neural networks are utilized to predict the strengths of interactions in AuNC–protein complexes on a coarse‐grained level, which are then optimized in Monte Carlo‐based structure search and refined to atomic‐scale structures. The training data is based on extensive molecular dynamics (MD) simulations of AuNC–protein complexes, and the validating MD simulations show the robustness of the predictions. This strategy can be generalized to any complexes of inorganic nanostructures and biomolecules provided that one generates enough data about the interactions, and the bioactive parts of the nanostructure can be coarse‐grained rationally.

## Graphical abstract

Graph theory‐based tools and artificial neural networks are used to estimate the strength of interactions between gold nanoclusters and blood proteins based on molecular dynamics (MD) simulation data. The method is able to predict favorable binding sites utilizing the estimated interactions. The sites are validated with half‐microsecond MD simulations, which demonstrate the stability of the predictions.

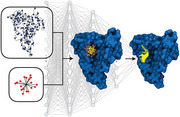

## 1 Introduction

Ligand‐stabilized metal nanoclusters (MNCs) are atomically precise metal nanoparticles with definite mass, structure, and chemical composition.^[^ ^1^ ^]^ Their metal core of 1–3 nm in diameter exhibits quantized electronic structure, and they are chemically stabilized by a molecular surface layer which is modifiable for functionalization and optimized biocompatibility, making them promising materials for novel applications in bioimaging, biosensing and nanomedicine as fluorescent markers, sensors and targeting drug carriers.^[^ ^2^ ^]^ Their ultrasmall size makes them amenable to atom‐scale modeling which may greatly help experimental efforts to design their properties for applications. However, modeling the dynamic interactions between MNCs and biomolecules is technically challenging due to the lack of suitable force fields and the wide range of time scales needed for discovering ensemble properties of MNC–biomolecule complexes.^[^ ^3^ ^]^

There is an increasing interest in using small, atomically precise, water‐soluble gold nanoclusters (AuNCs) for applications such as targeted drug delivery,^[^ ^4^ ^]^ biosensing,^[^ ^5^ ^]^ bioimaging,^[^ ^6^, ^7^ ^]^ and photodynamic therapy (PDT).^[^ ^8^, ^9^ ^]^ A common challenge for all‐atom simulations for these and other potential applications is to understand the atom‐scale interactions at the nano–bio interface, and their direct and indirect effects on the structure–function relations of the nano–bio complex. For instance, binding of an AuNC to a protein can enhance the fluorescence of the cluster^[^ ^10^, ^11^ ^]^ which is a desired effect in case one designs fluorescent protein markers. In some other cases, a wanted functionality of the nano–bio complex may be diminished or destroyed. One such example may be the still poorly known effects of a protein corona around a MNC, potentially affecting its targeting ability as a nano‐sized drug delivery vehicle, as observed in other nanomaterials.^[^ ^12^, ^13^ ^]^

Modeling how small molecules, molecular complexes, or nanoparticles interact with proteins is traditionally treated by docking algorithms. There are numerous published methods to model protein–protein,^[^ ^14^, ^15^, ^16^, ^17^, ^18^, ^19^ ^]^ ligand–protein,^[^ ^20^, ^21^, ^22^ ^]^ and nanomaterial–protein^[^ ^17^, ^23^, ^24^ ^]^ interactions. Docking algorithms commonly use experimentally determined crystal structures of the complexes to adjust docking parameters or to train underlying machine learning (ML) methods. Having crystal structures for fitting is naturally preferred as it guarantees that the model agrees with experimental observations. However, for some applications where experimental crystallographic data is limited or nonexistent, it is not an option. In this case, one has to rely on computationally generated model data. There has been a significant development work on methods able to utilize or even produce dynamic interaction data^[^ ^17^, ^24^ ^]^ but there is still an increasing demand for these tools. Being able to explore new protein binding sites and screen nano–bio interactions speeds up the discovery of new nanomaterials for biological applications in drug delivery, biosensing, and bioimaging, to name a few. In the case of nonconventional nanomaterials such as MNCs, it is desirable to have a method that could address its characteristics. The method presented in this study seeks to find an optimal balance between specificity and generality, an ability to address special characteristics of MNCs, and applicability to a range of interacting systems.

Here we introduce and validate a method named GraphBNC, designed to reliably predict the most optimal interaction sites between MNCs and proteins. As a case study, we selected AuNCs protected by *para*‐mercaptobenzoic acid (*p*‐MBA) or *para*‐mercaptobenzenesulfonic acid (*p*‐MBSA) with chemical formulae ${\mathrm{Au}}_{25}{\left(p-\mathrm{MBA}\right)}_{18}$, ${\mathrm{Au}}_{25}{\left(p-\mathrm{MBSA}\right)}_{18}$, and ${\mathrm{Au}}_{102}{\left(p-\mathrm{MBA}\right)}_{44}$ in combination with some of the most abundant proteins in blood plasma, such as albumin, apolipoprotein E, immunoglobulins G and E, and fibrinogen. The method does not require any pre‐existing experimental information on the target complex structure since the interactions are learned from training data produced by molecular dynamics (MD) simulations of the separate components of the complex. The validation is done by long MD simulations (up to 500 ns) of 13 complexes, where we demonstrate the success of the predictions for the optimal AuNC–protein interaction sites.

Although the method is constructed for a limited set of systems (gold nanoclusters and a set of blood proteins), it can be generalized to any nano–bio interface since it just requires the atomic coordinates of the system. If experimental data is available, it could be used in the model, but it is not required, which makes GraphBNC a particularly useful tool when empirical evidence is scarce. We envision that the method could be used to understand different molecular mechanisms at the nano–bio interface, such as the specific shape‐dependent inhibition behavior of nanoparticles,^[^ ^25^ ^]^ or the reversible control of protein corona formation using zwitterionic nanoparticle ligands,^[^ ^26^ ^]^ to name a few.

## 2 Results and Discussion

In this section, the GraphBNC method is introduced at a general level, showing how graphs, Feedforward Neural Network (FNN), and Monte Carlo (MC)‐based simulated annealing were combined to predict interactions between AuNCs and proteins. The method was used to generate a number of AuNC–protein complexes, which were validated by large‐scale MD simulations. The validation showed that predicted interaction sites were stable and the analysis showed the predicted interacting residues agreed well with the MD simulations.

### 2.1 GraphBNC Framework Design

**Figure 1:**
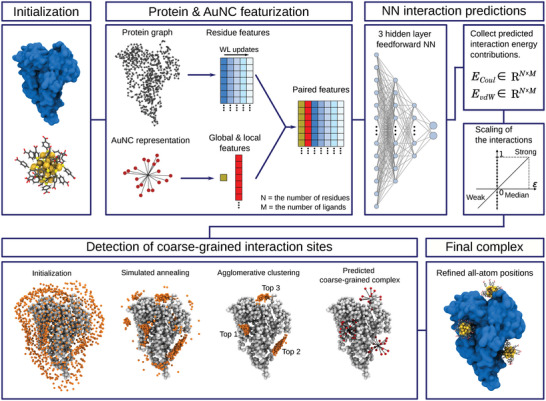
The GraphBNC framework consists of four steps: featurization, interaction energy predictions, coarse‐grained annealing placement, and refinement to all‐atom positions. In the coarse‐grained annealing, gray spheres represent the α‐carbons of the protein residues, while small orange spheres are the centers of the coarse‐grained nanocluster (AuNC). The centers were grouped with agglomerative clustering, and, in this case, the three largest ones were selected (Top 1–3). Three suggestions show corresponding coarse‐grained ligand head representations for single AuNCs that can be refined to the final all‐atom complexes.

The GraphBNC method consists of four stages: Protein and AuNC featurization, FNN estimation of the interaction strength, coarse‐grained simulated annealing prediction, and refinement of all‐atom positions. The general schematic of the method is illustrated in **Figure** **1**. The protein is presented as a graph, where alpha (α) carbons of the protein residues work as the nodes. If α‐carbons are adequately close to each other, they are connected via an edge. Every node contains a certain set of attributes, representing the chemical characteristics of the amino acid residues, their geometric environment, and their connectivity to other residues. A continuous Weissfeiler‐Lehman (WL) scheme was used to propagate information through the protein graph.^[^ ^27^, ^28^ ^]^ The functional groups of the protecting ligands of the AuNC are described with similar features accompanied with information about the shape and size of the AuNC as a whole entity (see details in Graph‐Based Representation Section). In order to predict contributions to the interaction energy, the ligand–residue pairs are formed, and their combined features are inputted into the FNN. Fivefold cross‐validation (CV) was used with a small separate validation set, hence the final predictions are averaged over five models trained with slightly different portions of data. The FNN is designed to predict Coulomb and van der Waals contributions of the AuNC–protein interaction energy. These energy estimates are scaled so that the strongest interaction gets the value of 1.0, the median is 0, and the rest are linearly scaled. The scaled interactions are introduced to the MC‐based simulated annealing scheme, which aims to maximize the interaction strength (minimize the energy) and minimize the geometric loss dictated by the loss function. Initially, annealing is done for a coarse‐grained system, where only α‐carbons of the protein residues and functional groups of the AuNC ligands are present. This generates statistics from which *n* most likely sites (*n* = 3 in this study) are chosen based on agglomerative clustering.^[^ ^29^ ^]^ The assumption is that the largest cluster of points is the statistically likeliest interaction site. For each of these sites, one AuNC configuration with the strongest predicted interactions is fine‐tuned to ensure that there are no overlapping atoms. This outputs *n* suggestions for the suitable placement of the AuNC on the given protein.

### 2.2 Training, Testing and Validation Using Small Nanocluster–Protein Complexes

To train and test the FNN part of the method, we used a dataset consisting of all‐atom MD simulation trajectories of bovine serum albumin (BSA, PDB ID: 4F5S),^[^ ^30^ ^]^ human serum albumin (HSA, PDB ID: 1N5U),^[^ ^31^ ^]^ the amino‐terminal domain of human apolipoprotein E (ApoE, PDB ID: 1LE2),^[^ ^32^ ^]^ and the fragment antigen‐binding (Fab) region of human immunoglobulin E (IgE, PDB ID: 7MLH)^[^ ^33^ ^]^ in complex with two different water‐soluble thiolate‐protected Au_25_ nanoclusters: ${\mathrm{Au}}_{25}{\left(p-\mathrm{MBA}\right)}_{18}$ and ${\mathrm{Au}}_{25}{\left(p-\mathrm{MBSA}\right)}_{18}$.^[^ ^34^, ^35^, ^36^ ^]^ During the training and testing phase, two hyperparameters were determined: the number of WL updates and the ligand–residue pair formation distance within the training data. The more WL updates are made, the more information the nodes contain about neighbors further away. The pair formation distance determines how far away pairs of α‐carbons and ligand heads are used as a training input. The averaged root mean squared errors (RMSEs) for the validation set are shown in Tables S1– S3 (Supporting Information). The corresponding averaged standard deviations (STDs) of the predicted interaction energies tell about how well the five CV models agree with each other. These STD evaluations are listed in Tables S4– S6 (Supporting Information). In order to prevent overfitting, the training of FNNs was stopped after 150 epochs. The progression of the training is shown in the learning curves visualized in Figure S1 (Supporting Information). Based on RMSE and STD analysis, two WL updates and pair formation distance of 10 Å were chosen as optimal hyperparameters. The final FNN validation results for different interaction energy terms are visualized in Figure S2 (Supporting Information).

**Figure 2:**
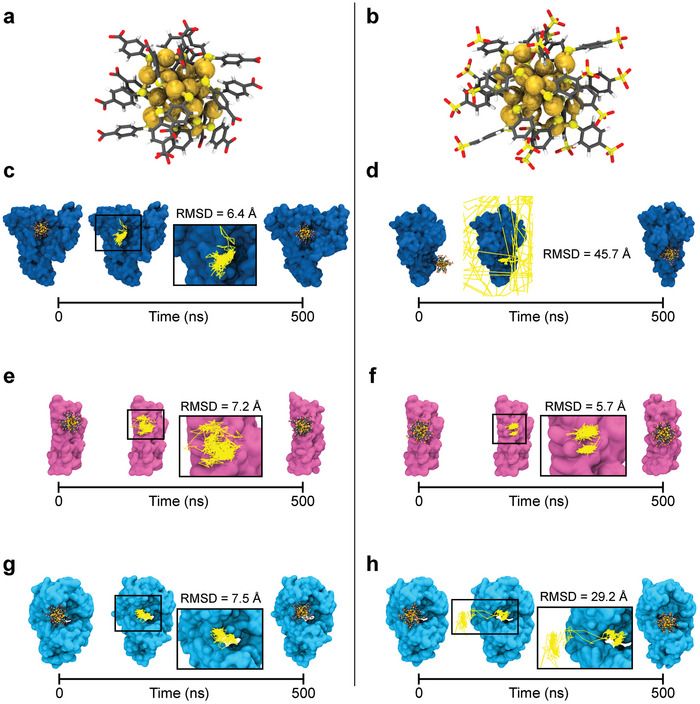
Validation of Au_25_NC‐protein complexes predicted by GraphBNC. On the left side: First and last snapshots from the molecular dynamics (MD) trajectory of the Top 1 binding sites of a) ${\mathrm{Au}}_{25}{\left(p-\mathrm{MBA}\right)}_{18}$ to c) human serum albumin (HSA), e) apolipoprotein E (ApoE), and g) immunoglobulin E (IgE). On the right side: First and last snapshots from the MD trajectory of the Top 1 binding sites of b) ${\mathrm{Au}}_{25}{\left(p-\mathrm{MBSA}\right)}_{18}$ to d) HSA, f) ApoE, and h) IgE. In each complex, the trajectory path of the AuNC is shown in yellow lines together with the averaged root mean square displacement (RMSD) with respect to its initial center of mass. Proteins are shown in different‐colored surface representation, while the metal core and ligand layer of AuNCs are shown as yellow spheres and sticks colored by atom type, respectively.

After training the FNN part of the GraphBNC, the overall performance of the method was validated. First top three sites were predicted for both ${\mathrm{Au}}_{25}{\left(p-\mathrm{MBA}\right)}_{18}$ and ${\mathrm{Au}}_{25}{\left(p-\mathrm{MBSA}\right)}_{18}$ on HSA, ApoE, and IgE proteins (Figure S3, Supporting Information) and the top 1 interaction site was chosen to be the representative case. The stability of the representative prediction was determined by running MD simulations of 500 ns for each complex (**Figure** **2**; see details in Molecular Dynamics Simulations of Nanocluster–Protein Complexes Section). The residues close to the AuNCs (cutoff distance = 4.0 Å) were followed throughout the MD trajectory (**Tables** **1** and **2**), and they were compared to the ones of the initial predicted site (Tables S7 and S8, Supporting Information). We determined how stable the predicted site was by analyzing how long the AuNCs stayed close to certain residues and how far the center of mass of the nanocluster fluctuated from the initial position on average.

**Table 1:** List of protein residues interacting with the nanocluster during the molecular dynamics simulation of the (${\mathrm{Au}}_{25}{\left(p-\mathrm{MBA}\right)}_{18}$)–protein complexes.

Complex	Interacting residues	Interaction time
(${\mathrm{Au}}_{25}{\left(p-\mathrm{MBA}\right)}_{18}$)–HSA	**LYS439 A**	**97.9%**
	**PRO441 A**	**93.3%**
	HID440 A	92.2%
	**LYS444 A**	**84.1%**
	LYS181 A	75.6%
	ARG160 A	60.7%
	ARG445 A	58.3%
	**PHE156 A**	**52.8%**
	CYS438 A	40.6%
(${\mathrm{Au}}_{25}{\left(p-\mathrm{MBA}\right)}_{18}$)–ApoE	LYS146 A	99.4%
	**ARG142 A**	**99.3%**
	LYS143 A	95.5%
	ARG145 A	78.1%
	ARG150 A	77.2%
	**SER139 A**	**61.5%**
	LEU149 A	51.7%
	ARG147 A	49.3%
(${\mathrm{Au}}_{25}{\left(p-\mathrm{MBA}\right)}_{18}$)–IgE	**LYS212 C**	**88.7%**
	LYS42 A	82.0%
	ASN161 C	74.7%
	**SER159 C**	**71.8%**
	THR157 C	71.5%
	**LYS207 C**	**69.8%**
	**ASN205 C**	**65.6%**
	**GLN111 C**	**63.4%**
	TYR108 C	48.3%
	TRP160 C	46.7%
	THR166 C	46.3%
	VAL158 C	45.2%
	**VAL5 C**	**44.5%**
	**GLN3 C**	**44.1%**
	**ASN203 C**	**42.3%**
(${\mathrm{Au}}_{25}{\left(p-\mathrm{MBA}\right)}_{18}$)–IgG	**LEU408 K**	**99.0%**
	**THR235 H**	**97.9%**
	**TYR306 H**	**97.9%**
	ARG311 H	95.0%
	**CYS236 H**	**94.0%**
	**PHE414 K**	**91.4%**
	**HID234 H**	**89.6%**
	**SER385 K**	**89.5%**
	**LYS256 H**	**87.2%**
	LYS350 K	71.7%
	**THR233 H**	**63.1%**
	LYS143 H	60.5%
	**PHE253 H**	**59.8%**
	**PHE251 H**	**52.6%**
	**VAL274 H**	**51.3%**
	GLU303 H	47.1%
	**VAL272 H**	**43.8%**

The results correspond to the analysis of 1,000 snapshots from each 500‐ns MD trajectory, and only protein residues interacting with the AuNC for more than 40% of the simulation time are listed. Protein residues at the GraphBNC‐predicted binding site are marked in bold. All interacting residues are specified by their residue ID and chain ID.

**Table 2:** List of protein residues interacting with the nanocluster during the molecular dynamics simulation of the (${\mathrm{Au}}_{25}{\left(p-\mathrm{MBSA}\right)}_{18}$)–protein complexes.

Complex	Interacting residues	Interaction time
(${\mathrm{Au}}_{25}{\left(p-\mathrm{MBSA}\right)}_{18}$)–HSA	ARG114 A	74.3%
	PRO113 A	74.1%
	LYS519 A	73.9%
	SER517 A	73.8%
	GLU518 A	72.3%
	VAL116 A	63.5%
	LEU179 A	56.0%
(${\mathrm{Au}}_{25}{\left(p-\mathrm{MBSA}\right)}_{18}$)–ApoE	**ARG142 A**	**100.0%**
	**LYS146 A**	**100.0%**
	ARG150 A	95.4%
	ARG145 A	78.1%
	**LEU149 A**	**67.9%**
	LYS143 A	65.6%
	ARG38 A	55.7%
	ARG147 A	50.7%
	ASP153 A	41.6%
(${\mathrm{Au}}_{25}{\left(p-\mathrm{MBSA}\right)}_{18}$)–IgE	**LYS212 C**	**61.8%**
	**ASN205 C**	**61.7%**
	LYS42 A	59.5%
	ASN161 C	57.3%
	GLN111 C	57.1%
	**LYS207 C**	**55.2%**
	**SER159 C**	**48.1%**
	**GLN3 C**	**45.2%**
	**VAL5 C**	**41.7%**
	TRP160 C	41.7%
(${\mathrm{Au}}_{25}{\left(p-\mathrm{MBSA}\right)}_{18}$)–IgG	ARG265 H	99.9%
	SER146 H	94.5%
	SER144 H	93.5%
	**LYS300 H**	**86.8%**
	GLY147 H	83.3%
	LYS298 H	77.3%
	LYS258 H	72.8%
	**LYS256 H**	**70.9%**
	GLY148 H	66.9%
	SER264 H	61.2%
	THR266 H	57.3%
	THR145 H	48.1%
	**LYS143 H**	**42.6%**

The results correspond to the analysis of 1,000 snapshots from each 500‐ns MD trajectory, and only protein residues interacting with the AuNC for more than 40% of the simulation time are listed. Protein residues at the GraphBNC‐predicted binding site are marked in bold. All interacting residues are specified by their residue ID and chain ID.

GraphBNC succeeded in predicting the interaction sites for ${\mathrm{Au}}_{25}{\left(p-\mathrm{MBA}\right)}_{18}$ with satisfying stability, as shown in Table 1 and Figure 2. In all (${\mathrm{Au}}_{25}{\left(p-\mathrm{MBA}\right)}_{18}$)–protein complexes, the *p*‐MBA ligands of the nanocluster interacted closely with the residues surrounding the GraphBNC‐predicted binding site and many of the initial interacting residues were maintained for about 90% of the simulation time. The number of residues naturally varies because GraphBNC handles the proteins and the AuNCs as rigid body objects, while in MD simulations, they are dynamic. This is why, for instance, only two predicted interacting residues are maintained during the MD simulation of (${\mathrm{Au}}_{25}{\left(p-\mathrm{MBA}\right)}_{18}$)–ApoE (Tables 1; Table S7, Supporting Information). Complexes with ${\mathrm{Au}}_{25}{\left(p-\mathrm{MBSA}\right)}_{18}$ were proven to be very dynamic, leading to varied residue lists in Table 2. ApoE and IgE predictions still reached a good agreement with MD simulations. When in complex with ApoE, the AuNC explored a small region (root mean square displacement (RMSD) = 5.7 Å) beyond the initial position. Two positively charged predicted residues (ARG142 and LYS146) were conserved during the whole MD trajectory, which were the main contributors to retaining the AuNC close to the initial position. Similarly, ${\mathrm{Au}}_{25}{\left(p-\mathrm{MBSA}\right)}_{18}$ started moving around the predicted interaction site on IgE, but during 165 ns (between 10–175 ns), it also explored an additional region –facilitated by the flexibility of elbow angle–, and then came back to the original site until the end of the simulation. This is seen as lower interaction times in the interacting residue lists (Table 2). These kinds of dynamic sites are challenging to any method. Originally, the site was a suitable‐sized pocket for the AuNC, but the deformation forced the AuNC to move. GraphBNC does not take this kind of dynamic behavior into account. However, the return of the ${\mathrm{Au}}_{25}{\left(p-\mathrm{MBSA}\right)}_{18}$ and the fact that it stayed in contact with the protein, proves the predicted interaction site to be favorable.

In addition, this observation is the first sign of the role that specific ligand chemistry might have on the antigen‐antibody reaction since higher flexibility of elbow angle is induced only by *p*‐MBSA ligands, which could interfere with the binding affinity of the Fab region to antigens.^[^ ^37^ ^]^

(${\mathrm{Au}}_{25}{\left(p-\mathrm{MBSA}\right)}_{18}$)–HSA, on the other hand, showed that the initial position obtained with GraphBNC was not accurate enough. The AuNC moved away from the protein as soon as the MD simulation started, and it remained in the solvent for 129 ns. For the rest of the simulation (371 ns), the AuNC was interacting with the protein, but in a region different from the predicted one. In order to analyze this complex in detail, we simulated another replicate of the system, and we observed a similar behavior (Figure S4, Supporting Information). The second replicate showed that the AuNC quickly detached from the protein, and it remained in the solvent for 48 ns. The stable interaction site was observed for 452 ns and was in agreement with the region detected in the first replicate. Thus, although the prediction was not as accurate as for the other systems, these findings demonstrate that GraphBNC also serves as a starting point for the detection of stable interaction sites between AuNCs and blood proteins under dynamic conditions.

We note that LYS and ARG are strongly present in all the predicted interaction sites. As positively charged residues, they establish strong electrostatic interactions with the negatively charged *p*‐MBA and *p*‐MBSA ligands (COO^−^ and ${\mathrm{SO}}_{3}^{-}$ head groups, respectively). This strongly suggests that the FNN is able to estimate the ligand–residue interactions reliably, and they are reflected correctly in the annealing process, leading the GraphBNC to converge into these interaction sites.

### 2.3 Extending Validation to Large Nanocluster–Protein Complexes

In order to validate the performance of the method beyond systems that were used for training, GraphBNC was applied to larger plasma proteins with ${\mathrm{Au}}_{25}{\left(p-\mathrm{MBA}\right)}_{18}$ and ${\mathrm{Au}}_{25}{\left(p-\mathrm{MBSA}\right)}_{18}$ nanoclusters. The chosen protein for this purpose was human immunoglobulin G (IgG, PDB ID: 1HZH).^[^ ^38^ ^]^ The method has not encountered IgG during the training/testing. Furthermore, IgG is a significantly larger protein than the ones used in training, with a molecular weight of ≈ 150 kDa versus 17, 66, and 47 kDa for ApoE, HSA, and IgE (Fab region), respectively.

**Figure 3:**
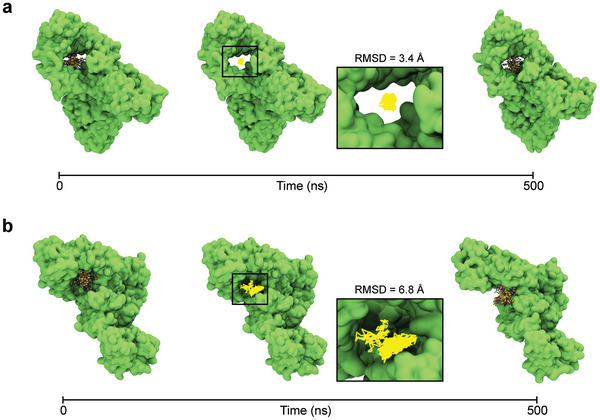
Validation of Au_25_NC–immunoglobulin G complexes predicted by GraphBNC. First and last snapshots from the molecular dynamics (MD) trajectory of the Top 1 binding sites of a) ${\mathrm{Au}}_{25}{\left(p-\mathrm{MBA}\right)}_{18}$ or b) ${\mathrm{Au}}_{25}{\left(p-\mathrm{MBSA}\right)}_{18}$ to immunoglobulin G (IgG). In each complex, the trajectory path of the AuNC is shown in yellow lines together with the averaged root mean square displacement (RMSD) with respect to its initial center of mass. Proteins are shown in green surface representation, while the metal core and ligand layer of AuNCs are shown as yellow spheres and sticks colored by atom type, respectively.

The IgG residues interacting with ${\mathrm{Au}}_{25}{\left(p-\mathrm{MBA}\right)}_{18}$ or ${\mathrm{Au}}_{25}{\left(p-\mathrm{MBSA}\right)}_{18}$ are listed at the end of the Tables 1 and 2, respectively. Tables S7 and S8 (Supporting Information) provide the full residue lists for the predicted interaction sites. GraphBNC fitted ${\mathrm{Au}}_{25}{\left(p-\mathrm{MBA}\right)}_{18}$ inside the fragment crystallizable (Fc) region of IgG, creating a highly stable complex, as illustrated in **Figure** **3**a. It is evident from Table 1 that the interacting residues fluctuate very little due to the strong interactions established in that confined space detected by GraphBNC. The initial position of ${\mathrm{Au}}_{25}{\left(p-\mathrm{MBSA}\right)}_{18}$ was also at the Fc region of IgG but slightly shifted toward the periphery due, in part, to the nature of its ligands (*p*‐MBSA ligands are a bit longer than *p*‐MBA). Thus, (${\mathrm{Au}}_{25}{\left(p-\mathrm{MBSA}\right)}_{18}$)–IgG complex was observed to be more dynamic than the aforementioned one (Figure 3b), but GraphBNC managed to predict strongly interacting LYS residues at the very close vicinity of the site, where ${\mathrm{Au}}_{25}{\left(p-\mathrm{MBSA}\right)}_{18}$ finally settled. These results also show how the ligand layer can promote the interaction of AuNCs with crucial sites of immunoglobulins for the regulation of innate and adaptive immunity, such as the Fc fragment,^[^ ^39^ ^]^ potentially inducing structural changes that can have a high impact on the immune response.^[^ ^40^ ^]^

**Figure 4:**
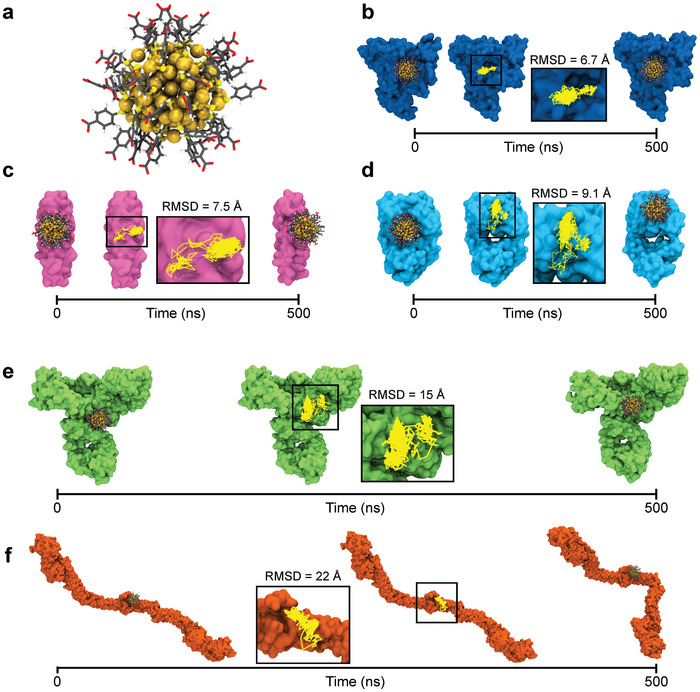
Validation of Au_102_NC–protein complexes predicted by GraphBNC. First and last snapshots from the molecular dynamics (MD) trajectory of the Top 1 binding sites of a) ${\mathrm{Au}}_{102}{\left(p-\mathrm{MBA}\right)}_{44}$ to b) human serum albumin (HSA), c) apolipoprotein E (ApoE), d) immunoglobulin E (IgE), e) immunoglobulin G (IgG), and f) Fibrinogen (Fib). In each complex, the trajectory path of the AuNC is shown in yellow lines together with the averaged root mean square displacement (RMSD) with respect to its initial center of mass. Proteins are shown in different‐colored surface representation, while the metal core and ligand layer of AuNCs are shown as yellow spheres and sticks colored by atom type, respectively.

The final stage of the validation was to use a larger AuNC than those used for training. We selected the well‐known ${\mathrm{Au}}_{102}{\left(p-\mathrm{MBA}\right)}_{44}$ nanocluster^[^ ^41^ ^]^ with all aforementioned proteins (HSA, ApoE, IgE, and IgG) and also human fibrinogen (Fib, PDB ID: 3GHG)^[^ ^42^ ^]^ (**Figure** **4**; Figure S5, Supporting Information). Complexes of ${\mathrm{Au}}_{102}{\left(p-\mathrm{MBA}\right)}_{44}$ with IgG and Fib are large and totally new for the method, and neither the AuNC nor the proteins were used to train the method. Thus, this enables the testing of the performance limits to its fullest.

**Table** **3** shows the analysis of the interaction site stability for ${\mathrm{Au}}_{102}{\left(p-\mathrm{MBA}\right)}_{44}$ in complex with HSA, ApoE, IgE, IgG, and Fib. Corresponding full residue lists for predicted interaction sites are presented in Table S9 (Supporting Information). With small proteins (i.e., HSA, ApoE, and IgE), GraphBNC predicts even more stable interaction sites than it did with ${\mathrm{Au}}_{25}{\left(p-\mathrm{MBA}\right)}_{18}$ and ${\mathrm{Au}}_{25}{\left(p-\mathrm{MBSA}\right)}_{18}$. The predicted interacting residues are maintained commonly over 90% of the simulation times, suggesting that the smaller curvature and larger ligand surface of Au_102_NC than its Au_25_ counterparts help create more interactions and then contribute to the more restricted movement of the proteins.

**Table 3:** List of protein residues interacting with the nanocluster during the molecular dynamics simulation of the (${\mathrm{Au}}_{102}{\left(p-\mathrm{MBSA}\right)}_{44}$)–protein complexes.

Complex	Interacting residues	Interaction time
(${\mathrm{Au}}_{102}{\left(p-\mathrm{MBA}\right)}_{44}$)–HSA	**LYS439 A**	**95.3%**
	**PRO441 A**	**93.7%**
	**LYS444 A**	**93.4%**
	HID440 A	89.1%
	GLU442 A	74.9%
	GLU294 A	62.9%
	LYS436 A	58.0%
	LYS274 A	56.9%
	LYS181 A	54.2%
(${\mathrm{Au}}_{102}{\left(p-\mathrm{MBA}\right)}_{44}$)–ApoE	**ARG147 A**	**100.0%**
	ARG150 A	99.4%
	**LYS143 A**	**99.2%**
	**HIE140 A**	**98.0%**
	SER139 A	90.8%
	ARG114 A	89.4%
	ARG142 A	88.9%
	LYS146 A	83.1%
	**ARG136 A**	**81.2%**
	ARG103 A	59.8%
	**ASP110 A**	**44.4%**
	VAL135 A	41.8%
(${\mathrm{Au}}_{102}{\left(p-\mathrm{MBA}\right)}_{44}$)–IgE	**GLN1 C**	**98.7%**
	**GLY57 A**	**98.3%**
	**GLN3 A**	**97.3%**
	PRO59 A	96.5%
	**THR56 A**	**95.8%**
	ARG61 A	94.6%
	SER60 A	92.5%
	**TYR108 C**	**81.8%**
	**ASP81 A**	**68.2%**
	GLN79 A	64.8%
	VAL58 A	42.8%
(${\mathrm{Au}}_{102}{\left(p-\mathrm{MBA}\right)}_{44}$)–IgG	ARG203 M	90.4%
	LYS284 H	69.5%
	**LYS336 H**	**66.7%**
	LYS332 H	42.8%
	PRO341 H	41.1%
	**SER334 H**	**40.9%**
(${\mathrm{Au}}_{102}{\left(p-\mathrm{MBA}\right)}_{44}$)–Fib	**LYS3 A**	**97.0%**
	ALA1 A	96.9%
	**LYS1 E**	**94.9%**
	CYS2 A	92.8%
	ALA1 D	89.3%
	**ASP4 A**	**71.8%**
	CYS2 D	56.0%
	**LYS3 D**	**50.3%**

The results correspond to the analysis of 1,000 snapshots from each 500‐ns MD trajectory, and only protein residues interacting with the AuNC for more than 40% of the simulation time are listed. Protein residues at the GraphBNC‐predicted binding site are marked in bold. All interacting residues are specified by their residue ID and chain ID.

One of the major AuNC displacements with respect to its initial position was observed in the (${\mathrm{Au}}_{102}{\left(p-\mathrm{MBA}\right)}_{44}$)–IgG complex. GraphBNC located the AuNC near the hinge region of IgG, a flexible tether that links the Fab and Fc portions of the protein. During the MD simulation, ${\mathrm{Au}}_{102}{\left(p-\mathrm{MBA}\right)}_{44}$ rolled on this site of the protein via interactions with LYS and ARG residues, mainly. From the predicted interaction sites, LYS and SER residues interacted the longest times: 66.7% and 40.9%, respectively. Even if the prediction was not perfect on this one, the method managed to bring the AuNC very close to its final site. Likewise, the interaction site detected between ${\mathrm{Au}}_{102}{\left(p-\mathrm{MBA}\right)}_{44}$ and Fib slightly differs from the predicted position. However, some LYS residues included in the predicted binding site, such as LYS3 (chain A) and LYS1 (chain E) remained interacting with the AuNC for more than 94% of the simulated time (Table 3). The AuNC was located at the so‐called central domain of Fib during the whole MD trajectory, and it was able to explore a wide region thanks to the flexibility of the LYS side chains.

The most drastic protein's structural changes were observed in this complex. ${\mathrm{Au}}_{102}{\left(p-\mathrm{MBA}\right)}_{44}$ interacting in the central domain of Fib can enhance the flexibility of Aα Bβ and γ chains (Figure 4f). These changes might alter the normal interaction of the central domain with the αC domains of Fib, which could lead to coagulation disorders.^[^ ^43^, ^44^ ^]^ This is a clear example of the necessity to build robust computational models for a preliminary nanomaterial risk assessment, especially now that the use of MNCs as diagnostic and therapeutic platforms is increasingly expanding.

## 3 Conclusion

In this work, the GraphBNC framework was introduced, and it was applied to predicting interaction sites for AuNCs on a set of blood proteins. It uses dynamic data from MD simulations to learn interaction energy contributions, making it especially useful for systems that have limited access to the crystal structures of the complexes. The predicted sites were confirmed by 0.5 microsecond MD simulations demonstrating that the method is capable of finding highly stable interaction sites. The movement of the AuNCs was traced from the MD simulations, showing that the AuNCs stayed in the imminent vicinity of the initial predicted sites. This was also evident from the lists of interacting residues. In conclusion, GraphBNC offers a new reliable tool for studying nanocluster–protein interactions, and it can initialize complexes for computations when experimental data about the structures is scarce.

## 4 Experimental Section

### Graph‐Based Representation

The proteins were represented as graph data structures, where α‐carbons were set as the nodes of the graphs. The nodes were connected via an edge if the corresponding α‐carbons were within 4.5 Å of each other. Every node contains a set of attributes, which can be divided into geometric, graph theoretical, and tabulated attributes in a similar way as in Refs. [17, 45]. These attributes are listed in **Table** **4**. Amino acids were divided into five types based on the nature of their side chains: hydrophobic (ALA, VAL, LEU, ILE, MET, PHE, TYR, TRP), polar uncharged (SER, THR, ASN, GLN), charged positive (ARG, HIS, LYS), charged negative (ASP, GLU), and “special” (CYS, SEC, GLY, PRO). These were represented as unit‐class vectors. Hydrophobicity values were taken from the study by Zviling et al., where amino acid hydrophobicities are optimized using various genetic algorithm approaches.^[^ ^46^ ^]^ The Define Secondary Structure of Proteins (DSSP) algorithm was used to classify residues based on the secondary structure they belong to.^[^ ^47^, ^48^ ^]^ Like amino acid typing, this was also presented as unit‐class vectors.

**Table 4:** Graph node attributes of the proteins. *N*_*x*_ are the counts of the element *x* atoms.

Attribute	Dimension
*N*_*C*_	1
*N*_*N*_	1
*N*_*O*_	1
*N*_*S*_	1
Molecular mass	1
Type of the amino acid	5
Hydrophobicity Ref. [46]	5
DSSP Refs. [47, 48]	8
Rel. ASA Ref. [49]	1
SASA (1.4 Å probe)	1
Acc. shell Ref. [50]	1
Pocketness Ref. [50]	1
Pocketness clust. Ref. [50]	5
*R*_*inacc*._^[^ ^50^ ^]^	1
OPD Chirality (5 & 7 n.n.) Ref. [51]	2
Ollivier–Ricci Refs. [52, 53, 54]	1
Forman–Ricci Refs. [53, 54, 55]	1
MFD Refs. [56, 57]	1
GNM Refs. [58, 59]	1
Features in total	39

Relative accessible surface area (Rel. ASA)^[^ ^49^ ^]^ and solvent accessible surface area (SASA) with 1.4 Å probe, accessible shell volume, and minimum inaccessible radius (*R*_*inacc*._)^[^ ^50^ ^]^ were used to measure how exposed residues are. There are six different Ghecom pocketness values in total. The first one is the main pocketness, and the remaining five were calculated using different clustering setups of the grid points within Ghecom software.^[^ ^50^ ^]^ Chirality is a key concept in biological interactions as, in many cases, systems are selective to the handedness. Hence, the local chirality was measured with the Osipov‐Pickup‐Dunmur (OPD) chirality index using the 5 and 7 nearest neighbors. Ollivier–Ricci and Forman–Ricci curvatures,^[^ ^52^, ^53^, ^54^, ^55^ ^]^ multifractal dimensionality (MFD, also called the “box‐counting dimension”),^[^ ^57^ ^]^ and Gaussian network modes (GNM)^[^ ^58^, ^59^ ^]^ were calculated from the graph architecture.

The protein graph is not only for calculating a few features but was also used to update node attributes using a continuous form of the Weisfeiler–Lehman (WL) scheme^[^ ^27^, ^28^ ^]^ with a similar idea as in reference.^[^ ^45^ ^]^ In this scheme, the node attributes are updated iteratively as:

(1) $$a^{i+1}\left(v\right)=\frac{1}{2}\left(a^{i}\left(v\right)+\frac{1}{\text{deg}(v)}\sum_{u\in N(v)}w\left(v,u\right)a^{i}\left(u\right)\right)$$

The superscript in Equation (1) refers to the iteration round and *a* ^*i*^(*v*) is the attribute of the node *v* from the *i*th iteration. The degree of the node is deg(*v*). N(v) is the set of neighbors of the node *v* and *w*(*v*, *u*) is the weight of the edge between nodes *v* and *u*. In this case, the edges are not weighted, thus all weights are one. This scheme increases the dimensionality of the final representation of a node with *N*_*WL*_ × *N*_*feat*_, where *N*_*WL*_ is the number of WL updates and *N*_*feat*_ is the number of initial features of the node.

**Figure 5:**
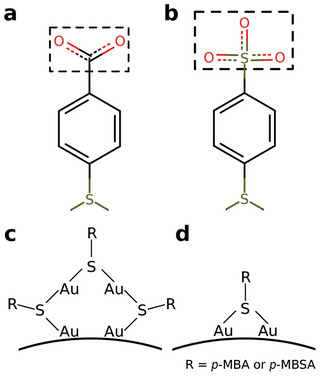
The dashed boxes highlight the head group of the a) *p*‐MBA and b) *p*‐MBSA ligands. The long protecting unit in panel c) demonstrates two types of locations for thiolate ligands based on the neighboring gold atoms. The ligand can be bound to a gold atom of the metallic core (on top of the curved line) and a gold atom part of the unit oligomer or both neighboring gold atoms could be a part of the unit as for the middle ligand. Panel d) shows the thiolate ligand on a bridge site.

Since biomolecular interactions of AuNCs are mainly dictated by the chemical nature of their ligands instead of their metallic core,^[^ ^60^ ^]^ the description focused on the protecting ligands. In this study, AuNCs contained two types of thiolate ligands: *p*‐MBA and *p*‐MBSA, which were featurized with six different descriptors. Three represent them with respect to other ligands, and three represent them on their own. The first descriptor is the minimum distance between the average position of the ligand head (*p*‐MBA: COO^−^; *p*‐MBSA: ${\mathrm{SO}}_{3}^{-}$) and neighboring heads (**Figure** **5**a,b). The second descriptor is a similar minimum distance but between the centers of the phenyl rings. In addition to these two distances, a categorical feature tells whether the ligand in question is a part of a π − π stacking. There are two minimum inaccessible radius measures: one averaged over all ligand atoms and one averaged over oxygen atoms. A categorical property also represents where the ligands are located on a protecting unit. The ligand can lie in the middle of the protecting unit, at the end, or at a bridge site (Figure 5c,d). However, Au_25_(SR)_18_ structures do not have any bridge site; thus, this value would be just a constant throughout the training dataset, and it is not contained as its own feature.

There are also a few global features representing the AuNC as a whole entity. There is a relative number of non‐hydrogen and gold atoms and a relative number of sulfur atoms with respect to the number of ligands. The size of the AuNC is measured with three relative properties. First, the spheres were formed into which the AuNC fits when only gold was included or when carbon atoms were also included. Then, the volume, surface area, and radii of the sphere were calculated after which the gold atom calculated values were divided by the carbon atom calculated ones. The last three values describe the shape of the AuNC. The moments of inertia of the AuNC were calculated and normalized them to sum up to one. As an example, if all inertia values are alike, then the AuNC is spherical. If one is significantly larger than two other ones, then the AuNC is oblate. If one value is significantly smaller than others, the shape is then cylindrical. All localized ligand features and global AuNC features are listed in **Table** **5**.

**Table 5:** Features of the nanocluster and its ligands.

	Attribute	Dimensionality
Nanocluster (global)	*N*_*non* − *H*_ /*N*	1
	*N*_*Au*_ /*N*	1
	*N*_*S*_ /*N*_*ligands*_	1
	*r*_*Au*_ /*r*_*C*_	1
	*A*_*Au*_ /*A*_*C*_	1
	*V*_*Au*_ /*V*_*C*_	1
	*J*_*i*_ / ∑_*j*_ *J*_*j*_	3
Total		9
Ligands (local)	min(*D*_*heads*_)	1
	min(*D*_*centers*_)	1
	π − π	1
	*R*_*inacc*._ (ligand)	1
	*R*_*inacc*._ (oxygen)	1
	position in Au‐SR unit	2 (3)
Total		7 (8)

Using the philosophy of the DeepRank‐GNN,^[^ ^16^ ^]^ the method uses two types of connectivity. First, there are the connections within the protein graphs as mentioned earlier. Second, pairs between the ligands of the AuNCs and the residues of the proteins are formed to serve as inputs for the machine‐learning engine. This can be considered as a bipartite graph having two distinct sets of nodes with connections formed between the sets. The pairwise representations consist of a global representation of the AuNC, a representation of a single ligand, and the WL updated representation of a protein graph node corresponding to the residue of interest, as shown in Figure 1. In this study, two WL updates were used.

### Neural Network Setup

Three hidden‐layer FNNs were used as the regression model. The number of neurons in the following hidden layer is half of the previous hidden layer (128, 64, and 32). A few different FNN sizes were tested with the next layer having half of the neurons of the previously layer. Accuracies did not differ significantly, hence relatively small network proved to be sufficient. Smaller NNs, contain less weight making them less prone to overfitting. The method is required to work on a range of different systems, hence smaller more robust models are desirable. As the activation functions, the Rectified Linear Unit (ReLU) was used. The learning rate was 0.001. The training was executed with Adam algorithm^[^ ^61^ ^]^ minimizing the Mean Absolute Error (MAE) of the sum of Coulombic and van der Waals parts of the AuNC–protein interaction energy, which were predicted separately. The batch size was 32. Input features were min‐max scaled into [0,1] interval, and output values were scaled into [− 1, 1]. The training was performed so that the method sums over all given ligand–residue pairs and tries to fit this to correspond the energy terms for the corresponding configuration.

### Annealing Procedure to Create Nanocluster–Protein Complexes

The AuNC was placed onto the protein in three phases. The first task is to run a Metropolis MC‐style simulated annealing algorithm to generate statistics. This was run for the coarse‐grained model, where only α‐carbons of the protein and the heads of the AuNC protecting ligands were considered. The loss function design was needed to address both the geometry of the interaction site and the estimated strength of the interaction. There can be a significant variation in pairwise interaction estimates. Thus, the total pairwise interaction energies were scaled linearly so that the median of the estimates is zero and the strongest interaction (the most negative energy) is one. This makes it easier to control how much the geometry and interaction strength contribute to the loss function. The annealing runs are initialized by rotating AuNCs randomly around their center of mass and bringing them from evenly distributed directions close to the protein.

The loss function used in simulated annealing is written as:

(2) $$\begin{array}{ccc} L & = & \sum_{j}^{N_{ligands}}\left[{\left(\frac{\theta }{d_{j}}\right)}^{4}-\left(\frac{\theta }{d_{j}}\right)-\frac{1}{2}\;\text{exp}\left(\frac{-{\left(d_{j}-d_{0}\right)}^{2}}{\sigma }\right)\right]\\ & - & \sum_{j}^{N_{close}}\left[\frac{1}{{\left(t_{j}-c\right)}^{4}}\right]-k\sum_{j}^{N_{pairs}}\phi_{j} \end{array}$$

(3) $$\theta =d_{0}\;4^{-1/3}$$

where *N*_*ligands*_ is the number of ligands, *N*_*close*_ is the number of α‐carbons within a certain distance from the center of the coarse‐grained AuNC, and *N*_*pairs*_ is the number of formed residue–ligand pairs. In the first summation, *d*_0_ is a parameter, which is the aimed distance between residue–ligand pairs. As the sum goes through the ligands, *d*_*j*_ is the minimum distance between the *j*th ligand and α‐carbons. The second summation prevents the algorithm from placing the AuNC inside the protein. Here, *c* is the minimum distance between the center of the AuNC and the ligand heads. The variable *t*_*j*_ is the distance between selected α‐carbons and the AuNC center. α‐carbons, for which (*t*_*j*_ − *c*) < 5.5 Å, are selected into the summation. In the third sum, ϕ_*i*_ are the scaled interaction strength estimates from the NN models. Pairs, which are within 5.5 Å from each other, are included in the sum. Parameter *k* is used to control the weight of the interaction. Here, it was set as one.

Random steps moving the AuNC representation were proposed by moving the AuNC toward a random direction and rotated around its center of mass according to some maximum rotation angle. The step sizes (both translation and rotation) are adjusted during the simulation so that the acceptances would stay between 40% and 60%. The probability of a step being accepted is based on the Metropolis question, which is written as

(4) $$P=\min\left(1,\text{exp}\left(\frac{-\Delta L}{k_{B}T}\right)\right)$$

Here, *k*_*B*_ is the Boltzmann constant, and *T* is the simulation temperature.

After obtaining an adequate amount of statistics, one has to select a few of the most favorable positions. This is done by agglomerative clustering.^[^ ^29^ ^]^ The center of mass points of the AuNCs are clustered, and *n* largest clusters are chosen for further selection. This relies on the assumption that the regions with the most points are statistically the likeliest and the most favorable for the interactions. From these sets of points, a single AuNC position was selected, which has the lowest interaction energy i.e., the interaction is the strongest. The selected position is still in the coarse‐grained form, and there might be overlapping atoms, therefore fine‐tuning with all atoms present is required. This could be done by hand or by running restricted simulated annealing. The restricted annealing is done the same way as the coarse‐grained one, but all atoms are present and the center of mass is required to stay within a certain distance from the initially determined site. The ligand‐residue pair contributes the interaction class sum in Equation (2) if at least one of their atoms is within 5.5 Å from each other. The parameter *d*_0_ in Equation (2) was set to 5.0 Å in the coarse‐grained annealing and then adjusted for the fine‐tuning annealing with all atoms present.

### Molecular Dynamics Simulations of Nanocluster–Protein Complexes

The predicted AuNC–protein complexes were subjected to all‐atom classical MD simulations using GROMACS 2023.3^[^ ^62^, ^63^ ^]^ with previously published AMBER‐compatible force field parameters for thiolate‐protected AuNCs.^[^ ^64^ ^]^ Each complex was placed in a periodic cubical box of TIP3P water,^[^ ^65^ ^]^ and 0.15 M NaCl was added to mimic the physiological conditions, including enough sodium ions to neutralize the system. Energy minimization was carried out with the steepest descent algorithm, followed by a two‐step equilibration procedure, which consisted of 10 ns in the NVT ensemble at 300 K and 10 ns in the NPT ensemble at 300 K and 1 bar pressure using the V‐rescale thermostat^[^ ^66^ ^]^ and the stochastic cell rescaling (C‐rescale) barostat.^[^ ^67^ ^]^ During equilibration, position restraints were applied to all nonhydrogen protein and AuNC atoms. Afterward, the restraints were removed and 500 ns of production MD was performed by keeping the temperature at 300 K and pressure at 1 bar using a timestep of 2.0 fs. The electrostatic interactions were modeled with the particle‐mesh Ewald (PME) method^[^ ^68^ ^]^ with a real‐space cutoff of 1.0 nm and a grid spacing of 0.12 nm, while the van der Waals interactions were modeled with Lennard–Jones potentials truncated at 1.0 nm. The SETTLE algorithm^[^ ^69^ ^]^ was used to constrain the internal degrees of freedom of the water molecules, and the bond lengths to hydrogens in the AuNC and protein were constrained with the linear constraint solver (LINCS) algorithm^[^ ^70^ ^]^ for improved performance.

Prior to the MD simulations of the AuNC–protein complexes, the crystal structure of each protein (HSA, chain A; ApoE, chain A; IgE, chains A and C; IgG, chains H, K, L, and M; Fib, chains A, B, C, D, E, and F) was prepared by adjusting the protonation states at pH 7.4 using the ProteinPrepare tool.^[^ ^71^ ^]^ For HSA, IgE, IgG, and Fib, the missing residues were added using PyMoL,^[^ ^72^ ^]^ and the missing loops in IgE and IgG were modeled with ModLoop.^[^ ^73^ ^]^ After that, each protein was fully relaxed by using the same procedure described above for energy minimization and equilibration phase, but extending the production MD until 1 µs.

Similarly, the production MD for each AuNC in water was run for 500 ns. The model structures of *p*‐MBA‐protected AuNCs were taken from previous studies,^[^ ^34^, ^35^ ^]^ while the model of ${\mathrm{Au}}_{25}{\left(p-\mathrm{MBSA}\right)}_{18}$ was built using GPAW software.^[^ ^74^ ^]^ The geometry was based on crystal structures of thiolate‐protected Au_25_ NCs^[^ ^75^, ^76^, ^77^ ^]^ and optimized with the Perdew–Burke–Erzerhof (PBE) exchange‐correlation functional^[^ ^78^ ^]^ using a 0.20 Å grid spacing and a force convergence criterion below 0.05 eV Å^−1^ per atom. The GROMACS topologies for the *p*‐MBSA ligands were obtained with ACPYPE code^[^ ^79^ ^]^ using a model system consisting of two gold atoms connected to two deprotonated ligands via a sulfur atom, as previously described.^[^ ^4^, ^64^ ^]^ This model was optimized at a B3LYP/LANL2DZ/W06 level of theory,^[^ ^80^ ^]^ and the electron density was used to calculate the electrostatic potential according to the Merz–Singh–Kollman scheme,^[^ ^81^, ^82^ ^]^ using Gaussian09.^[^ ^83^ ^]^ The atomic charges were fitted to the obtained potential in a two‐stage restrained electrostatic potential (RESP) fit procedure^[^ ^84^ ^]^ with AmberTools16,^[^ ^85^ ^]^ constraining the charges of Au atoms to zero.

### Data Collection

MD data was generated in three phases. The dataset was initialized with a sample of MD simulations of BSA with ${\mathrm{Au}}_{25}{\left(p-\mathrm{MBA}\right)}_{18}$ and ${\mathrm{Au}}_{25}{\left(p-\mathrm{MBSA}\right)}_{18}$. GraphBNC was then trained for the first time, and it was used to generate HSA–Au_25_ complexes. These structures were used to run MD simulations and then increase the training set. For the third phase, ApoE and IgE were added to the dataset used for the final training, testing, and validation. Complexes containing IgG, Fib, or ${\mathrm{Au}}_{102}{\left(p-\mathrm{MBA}\right)}_{44}$ were not used in training, but they were left for the final validation.

### Code Availability

GraphBNC is programmed using Python3 with Numpy,^[^ ^86^ ^]^ Scipy,^[^ ^87^ ^]^ Scikit‐Learn,^[^ ^88^ ^]^ Networkx,^[^ ^89^ ^]^ Atomic Simulation Environment (ASE),^[^ ^90^ ^]^ MDAnalysis,^[^ ^91^, ^92^ ^]^ Biopython,^[^ ^93^ ^]^ Prody^[^ ^94^ ^]^ and Tensorflow.^[^ ^95^ ^]^ Parallelization was implemented with MPI4Py.^[^ ^96^, ^97^, ^98^, ^99^ ^]^ 

## Conflict of Interest

The authors declare no conflict of interest.

## Supporting information

Supporting Information

## Data Availability

The code used in this study is available for download at https://doi.org/10.17011/jyx/dataset/96312 and up‐to‐date repository can be accessed via https://doi.org/10.17011/jyx/dataset/96311.

## References

[1] T. Tsukuda , H. Häkkinen , Protected Metal Clusters: From Fundamentals to Applications, Elsevier, Amsterdam, Netherlands, 2015.

[2] M. F. Matus , H. Häkkinen , Nat. Rev. Mater. 2023, 8, 372.

[3] S. Malola , H. Häkkinen , Nat. Commun. 2021, 12, 2197. 33850156 10.1038/s41467-021-22545-x PMC8044087

[4] M. F. Matus , S. Malola , H. Häkkinen , ACS Nanosci. Au 2021, 1, 47. 37102116 10.1021/acsnanoscienceau.1c00008 PMC10125177

[5] G. F. Combes , H. Fakhouri , C. Moulin , M. Girod , F. Bertorelle , S. Basu , R. Ladouce , M. P. Bakulić , Ž. S. Maršić , I. Russier‐Antoine , P. F. Brevet , Commun. Chem. 2021, 4, 1. 36697618 10.1038/s42004-021-00497-z PMC9814629

[6] C. J. Ackerson , P. D. Jadzinsky , G. J. Jensen , R. D. Kornberg , J. Am. Chem. Soc. 2006, 128, 2635. 16492049 10.1021/ja0555668

[7] C. J. Ackerson , P. D. Jadzinsky , J. Z. Sexton , D. A. Bushnell , R. D. Kornberg , Bioconjugate Chem. 2010, 21, 214. 10.1021/bc900135d PMC3113727 20099843

[8] R. Ho‐Wu , S. H. Yau , T. Goodson III , J. Phys. Chem. B 2017, 121, 10073. 29016137 10.1021/acs.jpcb.7b09442

[9] P. Aminfar , T. Ferguson , E. Steele , E. M. MacNeil , M. F. Matus , S. Malola , H. Häkkinen , P. N. Duchesne , H.‐P. Loock , K. G. Stamplecoskie , Nanoscale 2024, 16, 205. 10.1039/d3nr04793h 38051125

[10] J. Xie , Y. Zheng , J. Y. Ying , J. Am. Chem. Soc. 2009, 131, 888. 19123810 10.1021/ja806804u

[11] F. Bertorelle , K. D. Wegner , M. Perić Bakulić , H. Fakhouri , C. Comby‐Zerbino , A. Sagar , P. Bernadó , U. Resch‐Genger , V. Bonačić‐Kouteckỳ , X. Le Guével , R. Antoine , Chem. ‐ Eur. J. 2022, 28, 202200570. 10.1002/chem.202200570 35703399

[12] A. Salvati , A. S. Pitek , M. P. Monopoli , K. Prapainop , F. B. Bombelli , D. R. Hristov , P. M. Kelly , C. Åberg , E. Mahon , K. A. Dawson , Nat. Nanotechnol. 2013, 8, 137. 23334168 10.1038/nnano.2012.237

[13] Q. Dai , Y. Yan , C.‐S. Ang , K. Kempe , M. M. Kamphuis , S. J. Dodds , F. Caruso , ACS nano 2015, 9, 2876. 25712076 10.1021/nn506929e

[14] C. Dominguez , R. Boelens , A. M. J. J. Bonvin , J. Am. Chem. Soc. 2003, 125, 1731. 12580598 10.1021/ja026939x

[15] N. Renaud , C. Geng , S. Georgievska , F. Ambrosetti , L. Ridder , D. F. Marzella , M. F. Réau , A. M. J. J. Bonvin , L. C. Xue , Nat. Commun. 2021, 12, 7068. 34862392 10.1038/s41467-021-27396-0 PMC8642403

[16] M. Réau , N. Renaud , L. C. Xue , A. M. J. J. Bonvin , Bioinformatics 2022, 39, btac759. 10.1093/bioinformatics/btac759 PMC9805592 36420989

[17] M. Cha , E. S. T. Emre , X. Xiao , J.‐Y. Kim , P. Bogdan , J. S. VanEpps , A. Violi , N. A. Kotov , Nat. Comput. Sci. 2022, 2, 243. 38177552 10.1038/s43588-022-00229-w

[18] J. Tubiana , D. Schneidman‐Duhovny , H. J. Wolfson , Nat. Methods 2022, 19, 730. 35637310 10.1038/s41592-022-01490-7

[19] L. F. Krapp , L. A. Abriata , F. Cortés Rodriguez , M. Dal Peraro , Nat. commun. 2023, 14, 2175. 37072397 10.1038/s41467-023-37701-8 PMC10113261

[20] A. Volkamer , D. Kuhn , T. Grombacher , F. Rippmann , M. Rarey , J. Chem. Inf. Model. 2012, 52, 360. 22148551 10.1021/ci200454v

[21] A. Volkamer , D. Kuhn , F. Rippmann , M. Rarey , Bioinformatics 2012, 28, 2074. 22628523 10.1093/bioinformatics/bts310

[22] J. J. Clark , Z. J. Orban , H. A. Carlson , Sci. Rep. 2020, 10, 15856. 32985584 10.1038/s41598-020-72906-7 PMC7522209

[23] M. L. A. Hakkennes , F. Buda , S. Bonnet , J. Chem. Inf. Model. 2023, 63, 7816. 38048559 10.1021/acs.jcim.3c01582 PMC10751784

[24] J. C. Saldinger , M. Raymond , P. Elvati , A. Violi , Nat. Comput. Sci. 2023, 3, 393. 38177838 10.1038/s43588-023-00438-x

[25] S.‐H. Cha , J. Hong , M. McGuffie , B. Yeom , J. S. VanEpps , N. A. Kotov , ACS nano 2015, 9, 9097. 26325486 10.1021/acsnano.5b03247

[26] J. Mosquera , I. García , M. Henriksen‐Lacey , M. Martínez‐Calvo , M. Dhanjani , J. L. Mascarenñas , L. M. Liz‐Marzán , ACS nano 2020, 14, 5382. 32105057 10.1021/acsnano.9b08752 PMC7254833

[27] M. Togninalli , E. Ghisu , F. Llinares‐López , B. Rieck , K. Borgwardt , in Advances in Neural Information Processing Systems, (Eds.: H. Wallach , H. Larochelle , A. Beygelzimer , F. d' Alché‐Buc , E. Fox , R. Garnett ), vol. 32, Curran Associates, Inc., New York, 2019, pp. 6439–6449.

[28] N. Shervashidze , P. Schweitzer , E. J. Van Leeuwen , K. Mehlhorn , K. M. Borgwardt , J. Mach. Learn. Res. 2011, 12, 9.

[29] F. Nielsen , Introduction to HPC with MPI for Data Science, Springer International Publishing Switzerland, Cham, Switzerland 2016.

[30] A. Bujacz , Acta Crystallogr., Sect. D: Biol. Crystallogr. 2012, 68, 1278. 22993082 10.1107/S0907444912027047

[31] M. Wardell , Z. Wang , J. X. Ho , J. Robert , F. Ruker , J. Ruble , D. C. Carter , Biochem. Biophys. Res. Commun. 2002, 291, 813. 11866438 10.1006/bbrc.2002.6540

[32] C. Wilson , T. Mau , K. H. Weisgraber , M. R. Wardell , R. W. Mahley , D. A. Agard , Structure 1994, 2, 713. 7994571 10.1016/s0969-2126(00)00072-1

[33] K. Khatri , C. M. Richardson , J. Glesner , A. B. Kapingidza , G. A. Mueller , J. Zhang , C. Dolamore , L. D. Vailes , S. Wünschmann , R. S. Peebles Jr , M. D. Chapman , S. A. Smith , M. Chruszcz , A. Pomés , PNAS nexus 2022, 1, pgac054. 35799831 10.1093/pnasnexus/pgac054 PMC9248284

[34] K. Pyo , M. F. Matus , S. Malola , E. Hulkko , J. Alaranta , T. Lahtinen , H. Häkkinen , M. Pettersson , Nanoscale Adv. 2022, 4, 4579. 36425249 10.1039/d2na00487a PMC9606730

[35] E. Hulkko , T. Lahtinen , V. Marjomäki , E. Pohjolainen , V. Saarnio , K. Sokolowska , A. Ajitha , M. Kuisma , L. Lehtovaara , G. Groenhof , H. Häkkinen , M. Pettersson , Nanoscale Adv. 2021, 3, 6649. 36132657 10.1039/d1na00368b PMC9417352

[36] B. Zhang , Z. Wu , Y. Cao , Q. Yao , J. Xie , J. Phys. Chem. C 2020, 125, 489.

[37] M. L. Fernández‐Quintero , K. B. Kroell , M. C. Heiss , J. R. Loeffler , P. K. Quoika , F. Waibl , A. Bujotzek , E. Moessner , G. Georges , K. R. Liedl , Front. Mol. Biosci. 2020, 7, 609088. 33330636 10.3389/fmolb.2020.609088 PMC7732698

[38] E. O. Saphire , P. W. Parren , R. Pantophlet , M. B. Zwick , G. M. Morris , P. M. Rudd , R. A. Dwek , R. L. Stanfield , D. R. Burton , I. A. Wilson , Science 2001, 293, 1155. 11498595 10.1126/science.1061692

[39] A. Pincetic , S. Bournazos , D. J. DiLillo , J. Maamary , T. T. Wang , R. Dahan , B.‐M. Fiebiger , J. V. Ravetch , Nat. Immunol. 2014, 15, 707. 25045879 10.1038/ni.2939 PMC7430760

[40] T. Damelang , M. Brinkhaus , T. L. van Osch , J. Schuurman , A. F. Labrijn , T. Rispens , G. Vidarsson , Front. Immunol. 2024, 14, 1304365. 38259472 10.3389/fimmu.2023.1304365 PMC10800522

[41] P. D. Jadzinsky , G. Calero , C. J. Ackerson , D. A. Bushnell , R. D. Kornberg , Science 2007, 318, 430. 17947577 10.1126/science.1148624

[42] J. M. Kollman , L. Pandi , M. R. Sawaya , M. Riley , R. F. Doolittle , Biochemistry 2009, 48, 3877. 19296670 10.1021/bi802205g

[43] H. R. McPherson , C. Duval , S. R. Baker , M. S. Hindle , L. T. Cheah , N. L. Asquith , M. M. Domingues , V. C. Ridger , S. D. Connell , K. M. Naseem , H. Philippou , R. A Ajjan , R. AS Ariëns , Elife 2021, 10, e68761. 34633287 10.7554/eLife.68761 PMC8553339

[44] M. W. MOSESSON , J. TThromb. Haemost. 2005, 3, 1894. 10.1111/j.1538-7836.2005.01365.x 16102057

[45] A. Pihlajamäki , S. Malola , T. Kärkkäinen , H. Häkkinen , J. Phys. Chem. C 2023, 127, 14211.

[46] M. Zviling , H. Leonov , I. T. Arkin , Bioinformatics 2005, 21, 2651. 15797910 10.1093/bioinformatics/bti405

[47] W. Kabsch , C. Sander , Biopolymers 1983, 22, 2577. 6667333 10.1002/bip.360221211

[48] W. G. Touw , C. Baakman , J. Black , T. A. teBeek , E. Krieger , R. P. Joosten , G. Vriend , Nucleic. Acids Res. 2014, 43, D364. 25352545 10.1093/nar/gku1028 PMC4383885

[49] B. Rost , C. Sander , Proteins: Struct. Funct. Bioinf. 1994, 20, 216. 10.1002/prot.340200303 7892171

[50] T. Kawabata , Proteins: Struct. Funct. Bioinf. 2010, 78, 1195. 10.1002/prot.22639 19938154

[51] M. Osipov , B. Pickup , D. Dunmur , Mol. Phys. 1995, 84, 1193.

[52] C.‐C. Ni , Y.‐Y. Lin , J. Gao , X. David Gu , E. Saucan , in IEEE Conference on Computer Communications (INFOCOM), IEEE, New York, 2015, pp. 2758–2766.

[53] A. Samal , R. Sreejith , J. Gu , S. Liu , E. Saucan , J. Jost , Sci. rep. 2018, 8, 1. 29872167 10.1038/s41598-018-27001-3 PMC5988801

[54] C.‐C. Ni , Y.‐Y. Lin , F. Luo , J. Gao , Sci. Rep. 2019, 9, 1. 31292482 10.1038/s41598-019-46380-9 PMC6620345

[55] R. P. Sreejith , K. Mohanraj , J. Jost , E. Saucan , A. Samal , J. Stat. Mech.: Theory Exp. 2016, 2016, 063206.

[56] Y. Xue , P. Bogdan , Sci. rep. 2017, 7, 1. 28790321 10.1038/s41598-017-07209-5 PMC5548933

[57] K. Falconer , Fractal Geometry: Mathematical Foundations and Applications, John Wiley & Sons, Hoboken, New Jersey, 2004.

[58] I. Bahar , A. R. Atilgan , B. Erman , Fold. Des. 1997, 2, 173. 9218955 10.1016/S1359-0278(97)00024-2

[59] T. Haliloglu , I. Bahar , B. Erman , Phys. Rev. Lett. 1997, 79, 3090.

[60] A. A. Sousa , P. Schuck , S. A. Hassan , Nanoscale Adv. 2021, 3, 2995. 34124577 10.1039/d1na00086a PMC8168927

[61] D. P. Kingma , J. Ba , presented at *3rd Int. Conf. Learning Representations*, ICLR, San Diego, CA, USA, May 2015.

[62] M. J. Abraham , T. Murtola , R. Schulz , S. Páll , J. C. Smith , B. Hess , E. Lindahl , SoftwareX 2015, 1, 19.

[63] D. Van Der Spoel , E. Lindahl , B. Hess , G. Groenhof , A. E. Mark , H. J. C. Berendsen , J. Comput. Chem. 2005, 26, 1701. 16211538 10.1002/jcc.20291

[64] E. Pohjolainen , X. Chen , S. Malola , G. Groenhof , H. Häkkinen , J. Chem. Theory Comput. 2016, 12, 1342. 26845636 10.1021/acs.jctc.5b01053

[65] W. L. Jorgensen , J. Chandrasekhar , J. D. Madura , R. W. Impey , M. L. Klein , J. Chem. Phys 1983, 79, 926.

[66] G. Bussi , D. Donadio , M. Parrinello , J. Chem. Phys. 2007, 126, 1. 10.1063/1.2408420 17212484

[67] M. Bernetti , G. Bussi , J. Chem. Phys. 2020, 153, 11. 10.1063/5.0020514 32962386

[68] T. Darden , D. York , L. Pedersen , J. Chem. Phys. 1993, 98, 10089.

[69] S. Miyamoto , P. A. Kollman , J. Comput. Chem. 1992, 13, 952.

[70] B. Hess , H. Bekker , H. J. Berendsen , J. G. Fraaije , J. Comput. Chem. 1997, 18, 1463.

[71] G. Martínez‐Rosell , T. Giorgino , G. De Fabritiis , J. Chem. Inf. Model. 2017, 57, 1511. 28594549 10.1021/acs.jcim.7b00190

[72] L. Schrödinger , W. DeLano , Pymol, Schrödinger, Inc., New York 2020.

[73] A. Fiser , A. Sali , Bioinformatics 2003, 19, 2500. 14668246 10.1093/bioinformatics/btg362

[74] J. Enkovaara , C. Rostgaard , J. J. Mortensen , J. Chen , M. Dułak , L. Ferrighi , J. Gavnholt , C. Glinsvad , V. Haikola , H. Hansen , et al., J. Phys.: Condens.Matter 2010, 22, 253202. 21393795 10.1088/0953-8984/22/25/253202

[75] M. W. Heaven , A. Dass , P. S. White , K. M. Holt , R. W. Murray , J. Am. Chem. Soc. 2008, 130, 3754. 18321116 10.1021/ja800561b

[76] T. Dainese , S. Antonello , J. A. Gascon , F. Pan , N. V. Perera , M. Ruzzi , A. Venzo , A. Zoleo , K. Rissanen , F. Maran , ACS Nano 2014, 8, 3904. 24628268 10.1021/nn500805n

[77] M. A. Tofanelli , K. Salorinne , T. W. Ni , S. Malola , B. Newell , B. Phillips , H. Häkkinen , C. J. Ackerson , Chem. Sci. 2016, 7, 1882. 29899911 10.1039/c5sc02134k PMC5965251

[78] J. P. Perdew , K. Burke , M. Ernzerhof , Phys. Rev. Lett. 1996, 77, 3865. 10062328 10.1103/PhysRevLett.77.3865

[79] A. W. Sousa da Silva , W. F. Vranken , BMC Res. Notes 2012, 5, 1. 22824207 10.1186/1756-0500-5-367 PMC3461484

[80] F. Weigend , Phys. Chem. Chem. Phys. 2006, 8, 1057. 16633586 10.1039/b515623h

[81] U. C. Singh , P. A. Kollman , J. Comput. Chem. 1984, 5, 129.

[82] B. H. Besler , K. M. Merz Jr , P. A. Kollman , J. Comput. Chem. 1990, 11, 431.

[83] M. J. Frisch , et al., Gaussian 09, Revision E.01; Gaussian, Inc.: Wallingford, CT, 2013.

[84] C. I. Bayly , P. Cieplak , W. Cornell , P. A. Kollman , J. Phys. Chem. 1993, 97, 10269.

[85] D. Case , R. Betz , D. Cerutti , T. Cheatham , T. Darden , R. Duke , T. Giese , H. Gohlke , A. Goetz , N. Homeyer , AMBER 16; University of California: San Francisco, CA 2016.

[86] C. R. Harris , K. J. Millman , S. J. van der Walt , R. Gommers , P. Virtanen , D. Cournapeau , E. Wieser , J. Taylor , S. Berg , N. J. Smith , R. Kern , M. Picus , S. Hoyer , M. H. van Kerkwijk , M. Brett , A. Haldane , J. F. del Río , M. Wiebe , P. Peterson , P. Gérard‐Marchant , K. Sheppard , T. Reddy , W. Weckesser , H. Abbasi , C. Gohlke , T. E. Oliphant , Nature 2020, 585, 357. 32939066 10.1038/s41586-020-2649-2 PMC7759461

[87] P. Virtanen , R. Gommers , T. E. Oliphant , M. Haberland , T. Reddy , D. Cournapeau , E. Burovski , P. Peterson , W. Weckesser , J. Bright , S. J. van der Walt , M. Brett , J. Wilson , K. J. Millman , N. Mayorov , A. R. J. Nelson , E. Jones , R. Kern , E. Larson , C. J. Carey , İlhan Polat , Y. Feng , E. W. Moore , J. VanderPlas , D. Laxalde , J. Perktold , R. Cimrman , I. Henriksen , E. A. Quintero , C. R. Harris , et al., Nat. Methods 2020, 17, 261. 32015543 10.1038/s41592-019-0686-2 PMC7056644

[88] F. Pedregosa , G. Varoquaux , A. Gramfort , V. Michel , B. Thirion , O. Grisel , M. Blondel , P. Prettenhofer , R. Weiss , V. Dubourg , J. Vanderplas , A. Passos , D. Cournapeau , M. Brucher , M. Perrot , E. Duchesnay , J. Mach. Learn. Res. 2011, 12, 2825.

[89] A. A. Hagberg , D. A. Schult , P. J. Swart , in *Proc. of the 7th Python in Science Conf*., (Eds.: G. Varoquaux , T. Vaught , J. Millman ), Pasadena, CA USA, 2008, pp. 11–5.

[90] A. H. Larsen , J. J. Mortensen , J. Blomqvist , I. E. Castelli , R. Christensen , M. Dułak , J. Friis , M. N. Groves , B. Hammer , C. Hargus , E. D. Hermes , P. C. Jennings , P. B. Jensen , J. Kermode , J. R. Kitchin , E. L. Kolsbjerg , J. Kubal , K. Kaasbjerg , S. Lysgaard , J. B. Maronsson , T. Maxson , T. Olsen , L. Pastewka , A. Peterson , C. Rostgaard , J. Schiøtz , O. Schütt , M. Strange , K. S. Thygesen , T. Vegge , et al., J. Phys. Condens. Mat. 2017, 29, 273002. 10.1088/1361-648X/aa680e 28323250

[91] N. Michaud‐Agrawal , E. J. Denning , T. B. Woolf , O. Beckstein , J. Comput. Chem. 2011, 32, 2319. 21500218 10.1002/jcc.21787 PMC3144279

[92] R. J. Gowers , M. Linke , J. Barnoud , T. J. E. Reddy , M. N. Melo , S. L. Seyler , J. Domański , D. L. Dotson , S. Buchoux , I. M. Kenney , O. Beckstein , in *Proc. of the 15th Python in Science Conf*., (Eds.: S. Benthall , S. Rostrup ), Austin, Texas 2016, pp. 98–105.

[93] P. J. A. Cock , T. Antao , J. T. Chang , B. A. Chapman , C. J. Cox , A. Dalke , I. Friedberg , T. Hamelryck , F. Kauff , B. Wilczynski , M. J. L. de Hoon , Bioinformatics 2009, 25, 1422. 19304878 10.1093/bioinformatics/btp163 PMC2682512

[94] A. Bakan , L. M. Meireles , I. Bahar , Bioinformatics 2011, 27, 1575. 21471012 10.1093/bioinformatics/btr168 PMC3102222

[95] M. Abadi , A. Agarwal , P. Barham , E. Brevdo , Z. Chen , C. Citro , G. S. Corrado , A. Davis , J. Dean , M. Devin , S. Ghemawat , I. Goodfellow , A. Harp , G. Irving , M. Isard , Y. Jia , R. Jozefowicz , L. Kaiser , M. Kudlur , J. Levenberg , D. Mané , R. Monga , S. Moore , D. Murray , C. Olah , M. Schuster , J. Shlens , B. Steiner , I. Sutskever , K. Talwar , et al., TensorFlow: Large‐scale machine learning on heterogeneous systems, Software available from tensorflow.org 2015.

[96] L. Dalcín , R. Paz , M. Storti , J. Parallel Distr. Com. 2005, 65, 1108.

[97] L. Dalcín , R. Paz , M. Storti , J. D'Elía , J. Parallel Distr. Com. 2008, 68, 655.

[98] L. D. Dalcín , R. R. Paz , P. A. Kler , A. Cosimo , Adv. Water Resour. 2011, 34, 1124.

[99] L. Dalcín , Y.‐L. L. Fang , Comput. Sci. Eng. 2021, 23, 47.33967632

