# Supplementary material for: GraphBNC: Machine Learning‐Aided Prediction of Interactions Between Metal Nanoclusters and Blood Proteins
Source: Adv Mater. 2024 Sep 24;36(47):2407046. doi: 10.1002/adma.202407046 (PMC11586822; doi:10.1002/adma.202407046)
Supplement: Supplementary file 1 — Supporting Information [file ADMA-36-2407046-s001.pdf]

ADVANCED MATERIALS

Supporting Information

for *Adv. Mater.*, DOI 10.1002/adma.202407046

GraphBNC: Machine Learning-Aided Prediction of Interactions Between Metal Nanoclusters and Blood Proteins

*Antti Pihlajamäki, María Francisca Matus, Sami Malola and Hannu Häkkinen**

Supporting Information for "GraphBNC: Machine Learning-Aided Prediction of Interactions between Metal Nanoclusters and Blood Proteins"

Antti Pihlajamäki[†]

María Francisca Matus[†]

Sami Malola

*Hannu Häkkinen**

[†]equal contribution
Dr. A. Pihlajamäki
Department of Physics, Nanoscience Center, University of Jyväskylä, FI-40014 Jyväskylä, Finland
Dr. M. F. Matus
Department of Physics, Nanoscience Center, University of Jyväskylä, FI-40014 Jyväskylä, Finland
Dr. S. Malola
Department of Physics, Nanoscience Center, University of Jyväskylä, FI-40014 Jyväskylä, Finland
Prof. H. Häkkinen
Department of Physics, Nanoscience Center, University of Jyväskylä, FI-40014 Jyväskylä, Finland
Department of Chemistry, Nanoscience Center, University of Jyväskylä, FI-40014 Jyväskylä, Finland
Email Address: hannu.j.hakkinen@jyu.fi

1 Feedforward neural network validation

Table S1: Validation root-mean-squared error of the Coulombic interaction energy (kJ/mol) predicted by the 5-fold cross-validation FNN model.

WL update \ Pairing cutoff	5 Å	6 Å	7 Å	8 Å	9 Å	10 Å
0	67.28	65.68	63.62	57.62	55.39	53.09
1	66.26	65.40	62.56	56.80	54.05	51.28
2	66.88	65.04	63.00	57.09	54.14	**51.81**
3	67.37	65.92	63.26	57.46	54.61	54.00
4	67.44	66.18	63.75	58.25	55.93	53.60
5	67.77	66.20	63.41	58.76	55.66	52.85

The bold font value represents the one from the model used in the main method.

Table S2: Validation root-mean-squared error of the van der Waals interaction energy (kJ/mol) predicted by the 5-fold cross-validation FNN model.

WL update \ Pairing cutoff	5 Å	6 Å	7 Å	8 Å	9 Å	10 Å
0	18.61	17.89	16.27	15.68	15.53	15.28
1	18.41	17.62	16.09	15.64	15.36	15.73
2	18.59	17.54	16.31	15.71	15.32	**14.85**
3	18.55	17.72	16.17	15.48	15.33	16.08
4	18.50	17.44	16.28	15.66	15.47	14.98
5	18.54	17.58	16.29	15.72	15.39	14.97

The bold font value represents the one from the model used in the main method.

Table S3: Validation root-mean-squared error of the total interaction energies (Coulomb + van der Waals; kJ/mol) predicted by the 5-fold cross-validation FNN model.

WL update \ Pairing cutoff	5 Å	6 Å	7 Å	8 Å	9 Å	10 Å
0	68.83	67.06	63.46	56.93	53.40	51.79
1	67.78	66.54	62.06	56.11	52.25	49.54
2	68.57	66.24	62.69	56.42	52.28	**50.00**
3	68.95	66.87	62.90	56.58	52.78	54.04
4	69.05	67.43	63.19	57.37	54.07	51.39
5	69.36	67.41	62.96	57.96	53.89	50.83

The bold font value represents the one from the model used in the main method.

Table S4: Averaged standard deviations for the Coulombic interaction energy (kJ/mol) predicted by the 5-fold cross-validation FNN model.

WL update \ Pairing cutoff	5 Å	6 Å	7 Å	8 Å	9 Å	10 Å
0	18.54	18.40	19.25	17.95	18.12	18.99
1	19.84	19.16	18.07	19.87	20.39	18.66
2	17.76	19.39	17.87	21.53	25.96	**21.95**
3	19.38	16.72	19.25	19.80	26.74	19.76
4	18.54	20.22	19.48	19.54	17.83	24.62
5	16.35	20.67	18.53	21.95	21.39	23.43

The bold value represents the one from the model used in the main method.

Table S5: Averaged standard deviations for van der Waals interaction energy (kJ/mol) predicted by the 5-fold cross-validation FNN model.

WL update \ Pairing cutoff	5 Å	6 Å	7 Å	8 Å	9 Å	10 Å
0	4.26	4.49	4.44	5.14	4.39	4.41
1	5.06	5.11	4.67	4.68	6.27	4.39
2	4.77	5.10	5.46	5.86	6.70	**5.55**
3	5.40	5.97	4.47	5.35	5.07	5.47
4	5.11	4.74	4.77	5.64	5.59	7.51
5	4.59	4.99	5.19	6.14	7.02	5.64

The bold value represents the one from the model used in the main method.

Table S6: Averaged standard deviations for the total interaction energy (Coulomb + van der Waals; kJ/mol) predicted by the 5-fold cross-validation FNN model.

WL update \ Pairing cutoff	5 Å	6 Å	7 Å	8 Å	9 Å	10 Å
0	19.69	18.36	19.49	19.05	18.26	18.70
1	20.93	19.02	18.24	20.21	19.13	18.25
2	18.74	19.41	17.91	20.21	25.00	**21.08**
3	21.18	17.44	19.57	20.20	25.88	20.38
4	19.30	21.59	20.13	19.29	17.61	27.51
5	17.57	21.74	18.86	21.44	21.46	25.14

The bold value represents the one from the model used in the main method.

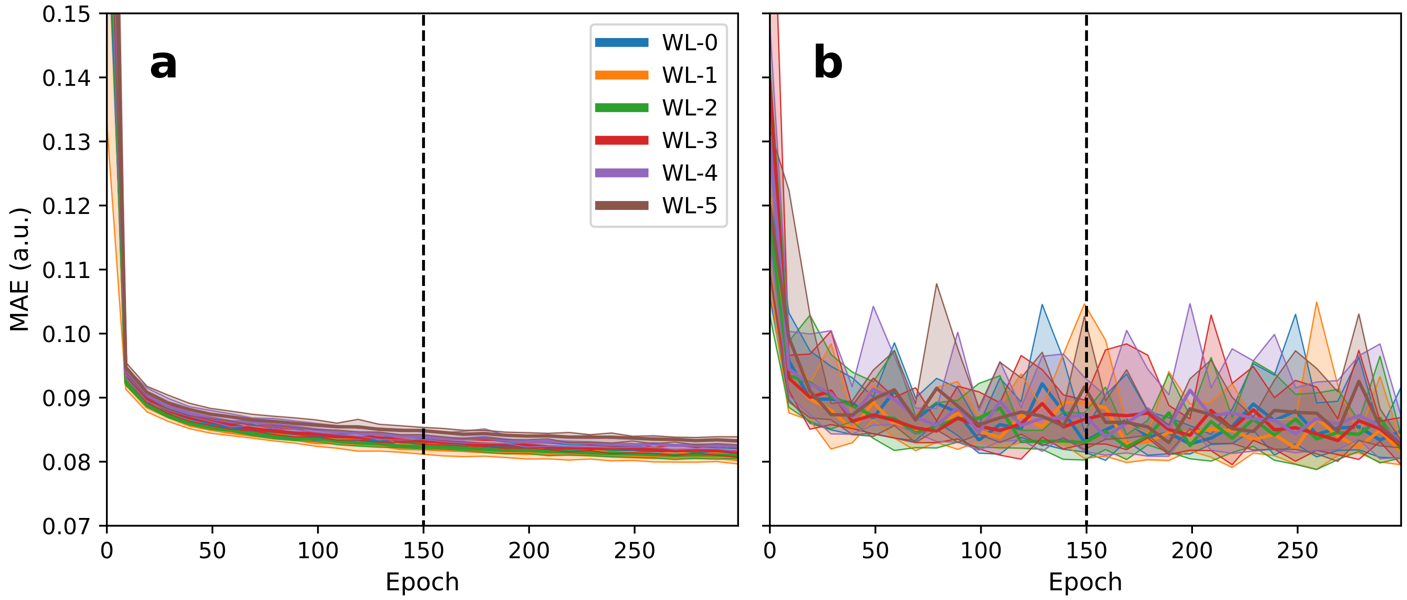

Figure S1: Learning curves of the FNN models with 10 Å pairing cutoff distances. The fast convergence on cross-validation sets is shown in the a) training error and b) test error. The mean absolute error (MAE) values are directly the error (in arbitrary units) that the training algorithm is minimizing with scaled training data. Thick lines are the average MAE, and the colored regions highlight the range of maximum and minimum values. The dashed vertical line shows where the training was considered adequate and where FNNs were taken for further usage.

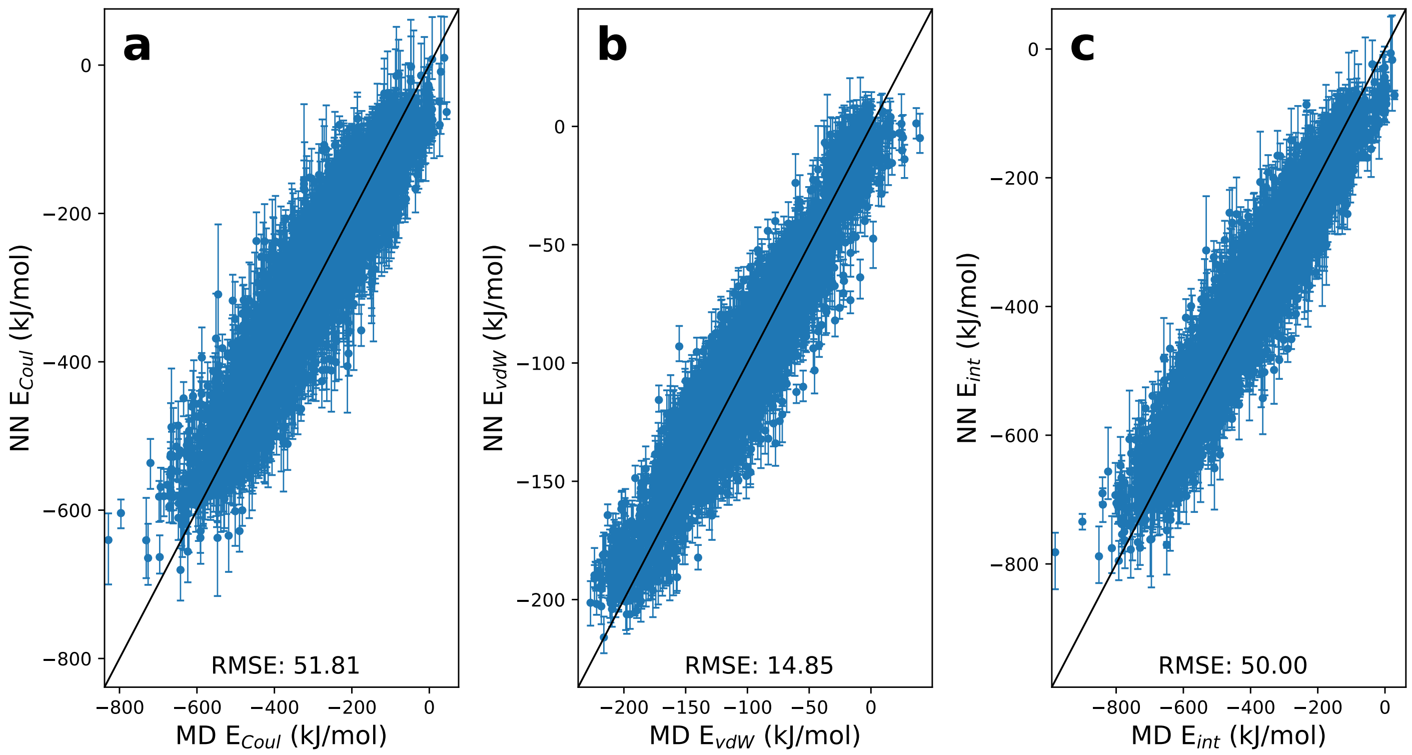

Figure S2: AuNC–protein interaction energies obtained from MD simulations versus AuNC–protein interaction energies predicted by the FNN model. The dot plots show the average predictions (with the maximum and minimum values) from 5-fold cross-validation models for a) the Coulombic interaction energy, b) the van der Waals interaction energy, and c) the total (Coulomb + van der Waals) interaction energy.

2 Predicted nanocluster–protein binding sites

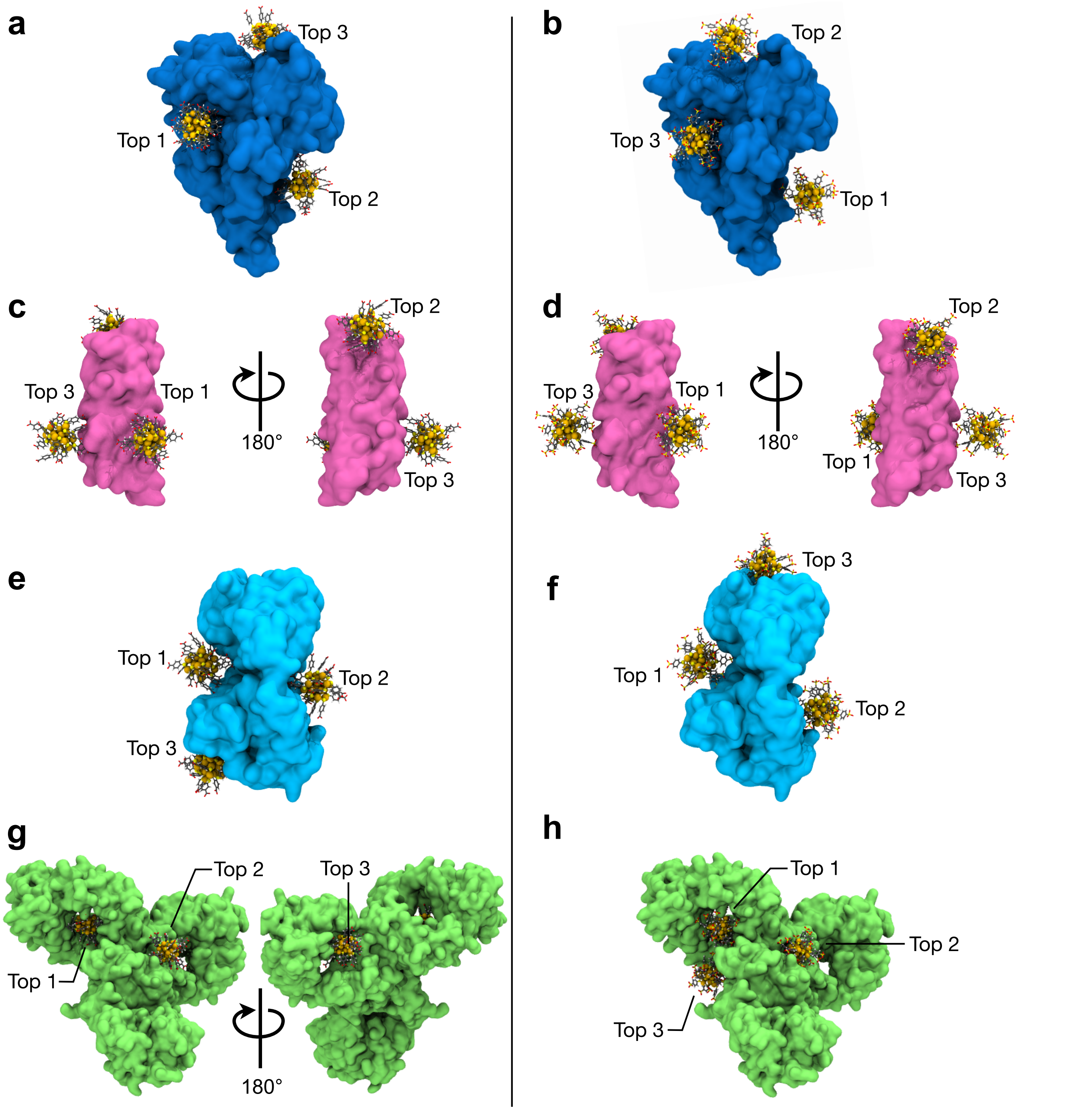

Figure S3: Au_{25}NC–protein binding sites predicted by GraphBNC. On the left side: Top 3 binding sites of $Au_{25}(p-\text{MBA})_{18}$ to a) human serum albumin (HSA), c) apolipoprotein E (ApoE), e) immunoglobulin E (IgE), and g) immunoglobulin G (IgG). On the right side: Top 3 binding sites of $Au_{25}(p-\text{MBSA})_{18}$ to b) HSA, d) ApoE, f) IgE, and h) IgG. Proteins are shown in different-colored surface representation, while the metal core and ligand layer of AuNCs are shown as yellow spheres and sticks colored by atom type, respectively.

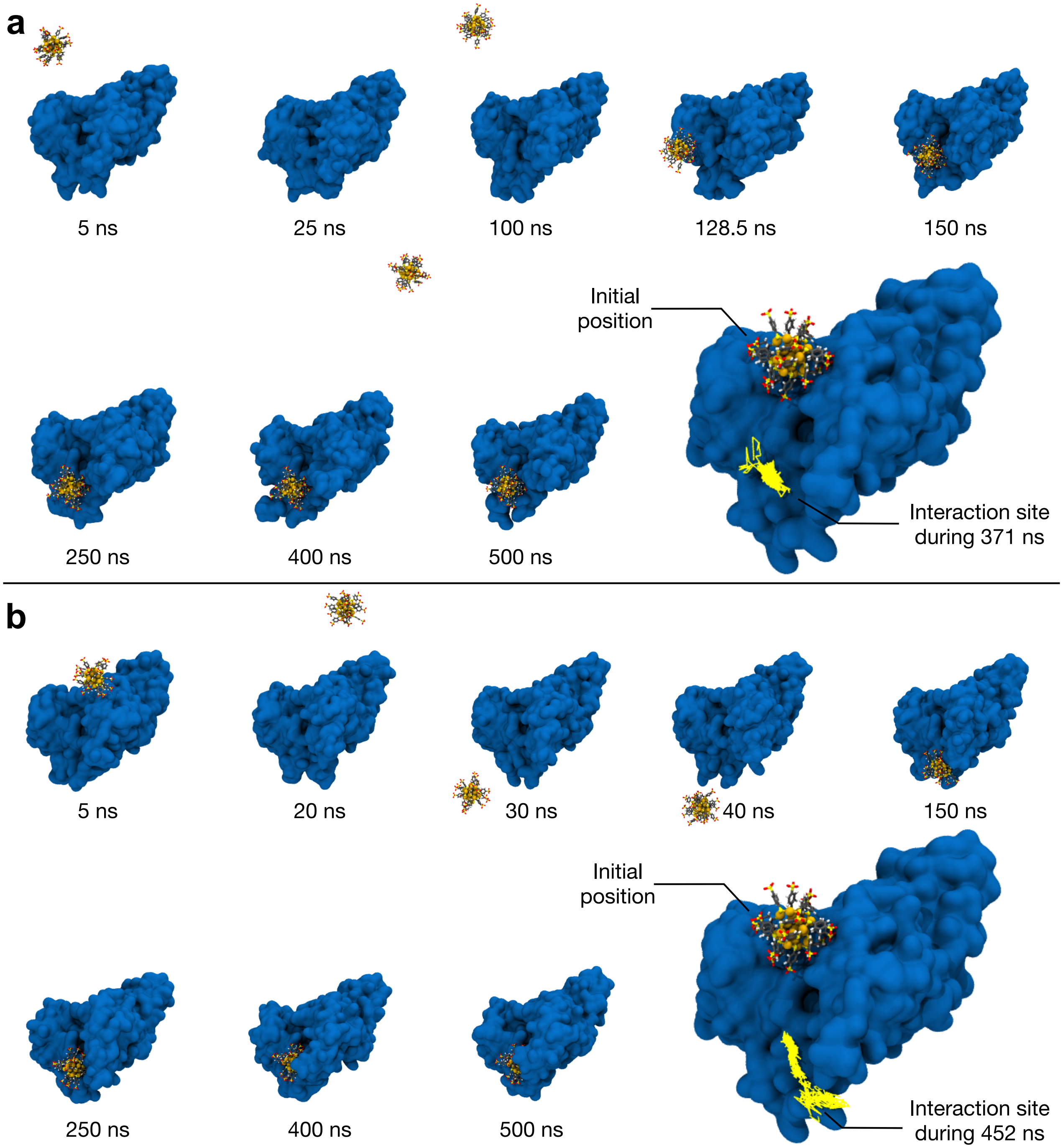

Figure S4: Observed $Au_{25}(p{-}MBSA)_{18}$–HSA binding sites. Snapshots of the interaction between $Au_{25}(p{-}MBSA)_{18}$ and human serum albumin (HSA) from a) replicate 1 and b) replicate 2 of 500 ns of molecular dynamics simulations. HSA is shown in blue surface representation, while the metal core and ligand layer of the AuNC are shown as yellow spheres and sticks colored by atom type, respectively. The differences between the predicted and observed binding sites are highlighted by showing the initial position of the AuNC (predicted by GraphBNC) together with its trajectory path (yellow lines) during the last 371 ns and 452 ns, for replicate 1 and replicate 2, respectively.

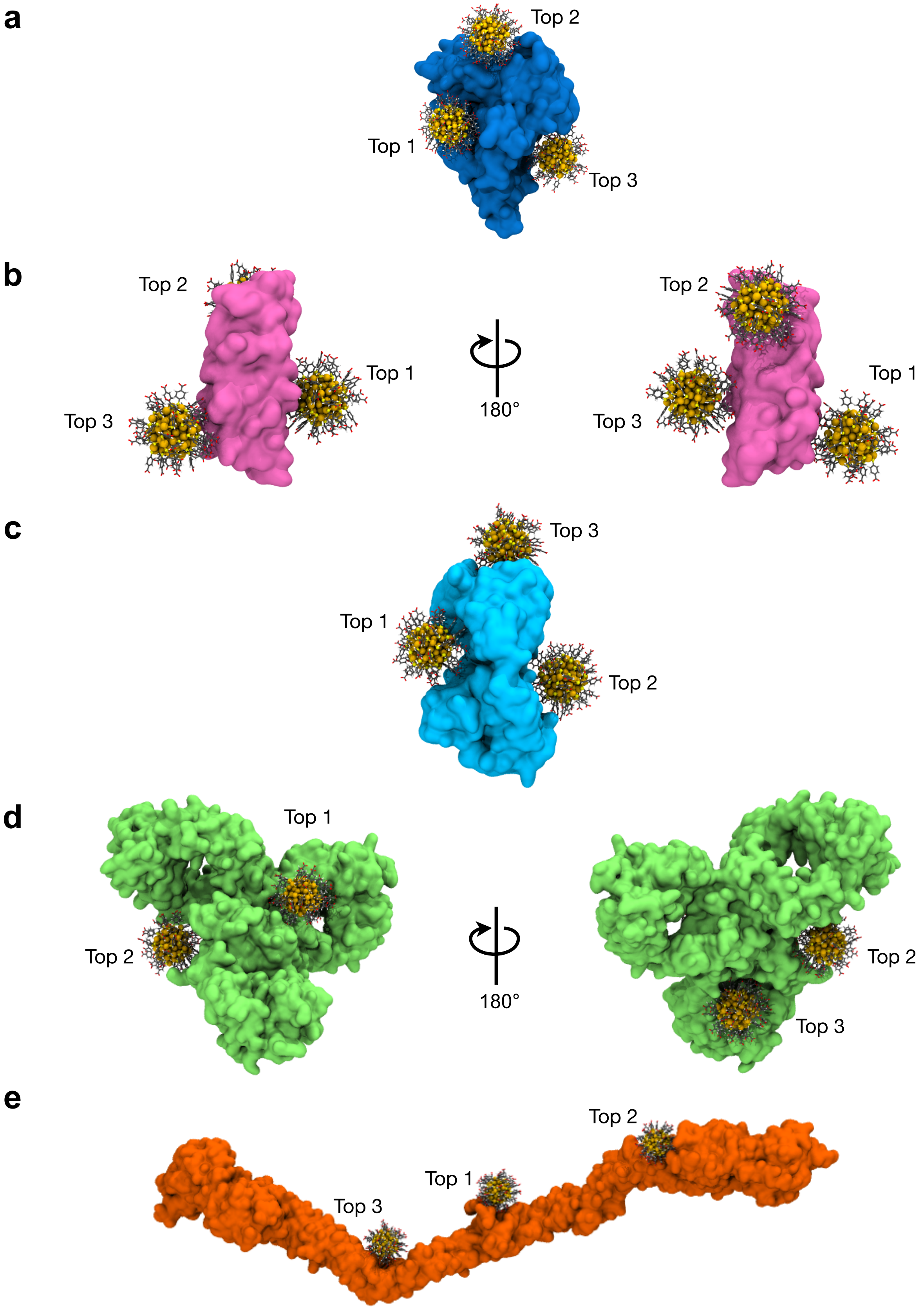

Figure S5: Au_{102}NC–protein binding sites predicted by GraphBNC. Top 3 binding sites of $Au_{102}(p-MBA)_{44}$ to a) human serum albumin (HSA), b) apolipoprotein E (ApoE), c) immunoglobulin E (IgE), d) immunoglobulin G (IgG), and e) fibrinogen (Fib). Proteins are shown in different-colored surface representation, while the metal core and ligand layer of AuNCs are shown as yellow spheres and sticks colored by atom type, respectively.

Table S7: List of GraphBNC-predicted protein residues interacting with $Au_{25}(p{-}MBA)_{18}$ and the interaction times from the molecular dynamics simulations.

Complex	Interacting residues	Interaction time
$(Au_{25}(p{-}MBA)_{18})$–HSA	LYS439 A	97.9%
	PRO441 A	93.3%
	LYS444 A	84.1%
	PHE156 A	52.8%
	GLU294 A	24.4%
	LYS276 A	11.0%
	VAL293 A	2.9%
	LYS274 A	1.7%
	GLU292 A	1.1%
	SER273 A	0.9%
	CYS289 A	0.8%
$(Au_{25}(p{-}MBA)_{18})$–ApoE	ARG142 A	99.3%
	SER139 A	61.5%
$(Au_{25}(p{-}MBA)_{18})$–IgE	LYS212 C	88.7%
	SER159 C	71.8%
	LYS207 C	69.8%
	ASN205 C	65.6%
	GLN111 C	63.4%
	VAL5 C	44.5%
	GLN3 C	44.1%
	ASN203 C	42.3%
	SER25 C	39.7%
	LYS23 C	24.1%
	ALA24 C	3.4%
$(Au_{25}(p{-}MBA)_{18})$–IgG	LEU408 K	99.0%
	THR235 H	97.9%
	TYR306 H	97.9%
	CYS236 H	94.0%
	PHE414 K	91.4%
	HID234 H	89.6%
	SER385 K	89.5%
	LYS256 H	87.2%
	THR233 H	63.1%
	HE253 H	59.8%
	PHE251 H	52.6%
	VAL274 H	51.3%
	VAL272 H	43.8%
	PRO405 H	19.1%
	TYR383 K	17.4%
	PRO406 H	12.1%
	SER249 H	6.0%
	ASP409 K	2.8%
	PRO384 K	2.4%
	PRO254 K	2.0%
	PHE251 K	1.7%
	LEU252 H	0.9%
	ILE346 K	0.1%
	VAL407 H	0.1%
	PHE253 K	0.1%
	PHE416 K	0.1%

The results correspond to the analysis of 1,000 snapshots from each 500-ns MD trajectory. All interacting residues are specified by their residue ID and chain ID.

Table S8: List of GraphBNC-predicted protein residues interacting with $Au_{25}(p{-}MBSA)_{18}$ and the interaction times from the molecular dynamics simulations.

Complex	Interacting residues	Interaction time
$(Au_{25}(p{-}MBSA)_{18})$–HSA	LYS500 A	0.3%
	GLU501 A	0.1%
	ASN503 A	0.1%
$(Au_{25}(p{-}MBSA)_{18})$–ApoE	ARG142 A	100.0%
	LYS146 A	100.0%
	LEU149 A	67.9%
	SER139 A	34.6%
$(Au_{25}(p{-}MBSA)_{18})$–IgE	LYS212 C	61.8%
	ASN205 C	61.7%
	LYS207 C	55.2%
	SER159 C	48.1%
	GLN3 C	45.2%
	VAL5 C	41.7%
	GLN6 C	36.8%
	THR157 C	35.8%
	SER25 C	32.3%
	ASN203 C	30.8%
	GLY26 C	28.9%
	LYS23 C	11.1%
	GLY8 C	9.5%
	SER7 C	6.6%
$(Au_{25}(p{-}MBSA)_{18})$–IgG	LYS300 H	86.8%
	LYS256 H	70.9%
	LYS143 H	42.6%
	VAL313 H	32.1%
	THR270 H	19.7%
	LYS402 H	11.6%
	ARG311 H	10.7%
	SER410 K	7.4%
	VAL274 H	7.0%
	THR235 H	6.8%
	HID234 H	6.0%
	LEU408 K	4.7%
	GLN305 H	4.0%
	TYR306 H	3.1%
	VAL272 H	1.0%
	PHE414 K	0.6%
	ASP409 K	0.6%
	PHE253 H	0.3%
	PRO254 H	0.3%

The results correspond to the analysis of 1,000 snapshots from each 500-ns MD trajectory. All interacting residues are specified by their residue ID and chain ID.

Table S9: List of GraphBNC-predicted protein residues interacting with $Au_{102}(p{-}MBA)_{44}$ and the interaction times from the molecular dynamics simulations.

Complex	Interacting residues	Interaction time
$(Au_{102}(p{-}MBA)_{44})$–HSA	LYS439 A	95.3%
	PRO441 A	93.7%
	LYS444 A	93.4%
	LYS276 A	27.4%
	PHE156 A	8.6%
	ARG160 A	5.3%
	ALA163 A	2.8%
$(Au_{102}(p{-}MBA)_{44})$–ApoE	ARG147 A	100.0%
	LYS143 A	99.2%
	HIE140 A	98.0%
	ARG136 A	81.2%
	ASP110 A	44.4%
	GLN117 A	4.4%
$(Au_{102}(p{-}MBA)_{44})$–IgE	GLN1 C	98.7%
	GLY57 A	98.3%
	GLN3 A	97.3%
	THR56 A	95.8%
	TYR108 C	81.8%
	ASP81 A	68.2%
	GLY26 C	5.9%
	ASN45 A	2.1%
	LEU165 C	0.2%
$(Au_{102}(p{-}MBA)_{44})$–IgG	LYS336 H	66.7%
	SER334 H	40.9%
	LYS220 H	19.4%
	LYS146 M	17.5%
	ASP222 H	8.9%
	THR219 H	4.6%
	GLU282 H	3.8%
	VAL221 H	3.0%
	THR198 M	2.3%
	ASP280 H	1.4%
	LYS150 M	1.0%
	LEU155 M	0.9%
	GLU279 H	0.7%
	PRO205 M	0.3%
	SER204 M	0.3%
$(Au_{102}(p{-}MBA)_{44})$–Fib	LYS3 A	97.0%
	LYS1 E	94.9%
	ASP4 A	71.8%
	LYS3 D	50.3%
	SER5 A	29.4%
	ASP4 D	21.3%
	ARG24 D	7.1%
	PRO13 B	3.6%
	ASP6 A	1.0%
	ASP4 E	0.4%
	PRO3 E	0.1%
	ALA2 E	0.1%

The results correspond to the analysis of 1,000 snapshots from each 500-ns MD trajectory. All interacting residues are specified by their residue ID and chain ID.
